# Re‐Thinking Electronic Circuit Design Through Emerging Reconfigurable Field Effect Transistors

**DOI:** 10.1002/advs.78121

**Published:** 2026-10-14

**Authors:** Jens Trommer, Juan P. Martinez, Yuxuan He, Giulio Galderisi, Katrin Pingen, Eduardo Sergio Oliveros‐Mata, Lisa Tellis, Cigdem Cakirlar, Christoph Beyer, Brojogopal Sapui, Joachim Knoch, Klaus Hofmann, Sandro Carrara, Eugenio Cantatore, Max C. Lemme, Ian O'Connor, Akash Kumar, Andre Heinzig, Thomas Mikolajick, Walter M. Weber

**Affiliations:** ^1^ Namlab gGmbH Dresden Saxony Germany; ^2^ Chair of Nanoelectronics Technische Universität Dresden Dresden Germany; ^3^ Institute of Semiconductor Electronics RWTH Aachen Aachen Germany; ^4^ Integrated Electronic Systems Lab TU Darmstadt Darmstadt Germany; ^5^ Bio/CMOS Interfaces Lab Institute of Electrical and Micro Engineering École Polytechnique Fédérale de Lausanne (EPFL) Neuchâtel Switzerland; ^6^ Department of Electrical Engineering Eindhoven University of Technology Eindhoven Netherlands; ^7^ Chair of Electronic Devices, RWTH Aachen University AMO GmbH, Advanced Microelectronic Center Aachen Aachen Germany; ^8^ Centrale Lyon, INSA Lyon, CNRS Ecole Central Lyon Ecully France; ^9^ Chair for Embedded Systems Ruhr‐University Bochum Bochum Germany; ^10^ Institute of Solid State Electronics TU Wien Vienna Austria

**Keywords:** bio‐sensors, circuit design, cryogenic temperatures, hardware security, logic‐in‐memory, reconfigurable field effect transistors, van‐der‐waals materials

## Abstract

Reconfigurable field‐effect transistors, short RFETs, are a unique class of emerging electronic devices that merge *n*‐type, *p*‐type, and ambipolar operation modes into a single element. Unlike conventional MOSFETs, the dominant carrier type in RFETs is purely defined by electrostatics and can be dynamically changed at runtime. This enables new design possibilities by enabling post‐fabrication functionality definition, thereby increasing circuit density and adaptability without requiring additional feature‐size scaling. However, the added value of the devices requires a reassessment of classical design paradigms to unlock their full potential. This paper aims to provide a comprehensive review of the state of the art in RFET circuit design. Starting from the underlying device physics and material systems, such as group‐IV and two‐dimensional (2D) materials, the manifold circuit design possibilities in digital, analog, and post‐Shannon computing are introduced. We highlight how RFETs aim to enable simplified, energy‐efficient, and reusable hardware for edge computing, hardware security, bio‐sensing, and cryogenic electronics. Thereby, device‐ and circuit‐level results are exemplified by measurements of transistors fabricated in an industrial 22 nm FDSOI technology. The paper also provides an overview of future key challenges in reliability, BEOL integration, and electronic design automation, offering an outlook on the path towards scalable, sustainable reconfigurable electronics.

## Graphical abstract

Graphical illustration of reconfigurable field effect transistor (RFET) operation modes, including *n*‐type, *p*‐type, and ambipolar transport, as well as their areas of application discussed in this review: digital circuit design, analog circuit design, post‐Shannon computing, and sensing.

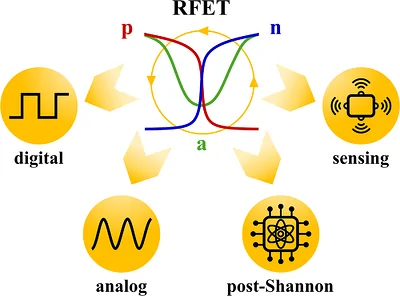

## 1 Introduction

Roughly 100 years after its inception, the metal‐oxide‐semiconductor field‐effect transistor (MOSFET) remains the dominant electron device in electronic systems worldwide. For decades, continued scaling of device dimensions enabled successive technology generations with higher integration density, faster operation, and improved energy efficiency. However, as scaling has progressed below the 10 nm node, many of the benefits traditionally associated with CMOS scaling have begun to diminish. Although transistor densities continue to increase at advanced technology nodes, performance improvements have become increasingly incremental and are accompanied by growing design complexity. At the same time, energy consumption, fabrication costs, and challenges related to reliability and variability continue to rise. These limitations are particularly relevant in view of the rapidly growing computational demands of artificial‐intelligence (AI) workloads, which increasingly challenge the performance and energy efficiency of conventional hardware.

Consequently, alternative approaches for improving circuit‐ and system‐level functionality and efficiency beyond conventional device scaling have attracted significant interest. Prominent directions include heterogeneous integration using chiplets and novel computing architectures that mitigate the von Neumann bottleneck by bringing computation and memory into closer proximity. Both approaches can benefit from the integration of emerging device technologies, including thin‐film transistors, non‐volatile memory elements, and electro‐optical components, into conventional electronic systems.

A fundamentally different emerging device concept, the reconfigurable field‐effect transistor (RFET), aims to extend functionality at the device level and thereby change how conventional CMOS circuits are designed [1]. While the polarity of conventional MOSFETs is fixed during fabrication, the functionality of an RFET can be electrically configured after fabrication [2]. This additional degree of freedom opens a new circuit‐design space in which a single device can assume multiple functions, offering the potential to increase functionality per unit chip area without relying solely on further dimensional scaling. Such fine‐grain reconfigurable circuits provide a compelling energy‐efficient alternative to state‐of‐the‐art high‐performance application‐specific integrated circuit (ASIC) designs with fixed functionality, and to established coarse‐grain reconfigurable field‐programmable‐gate‐arrays (FPGAs) with mediocre energy‐efficiency (Figure 1a).

**FIGURE 1:**
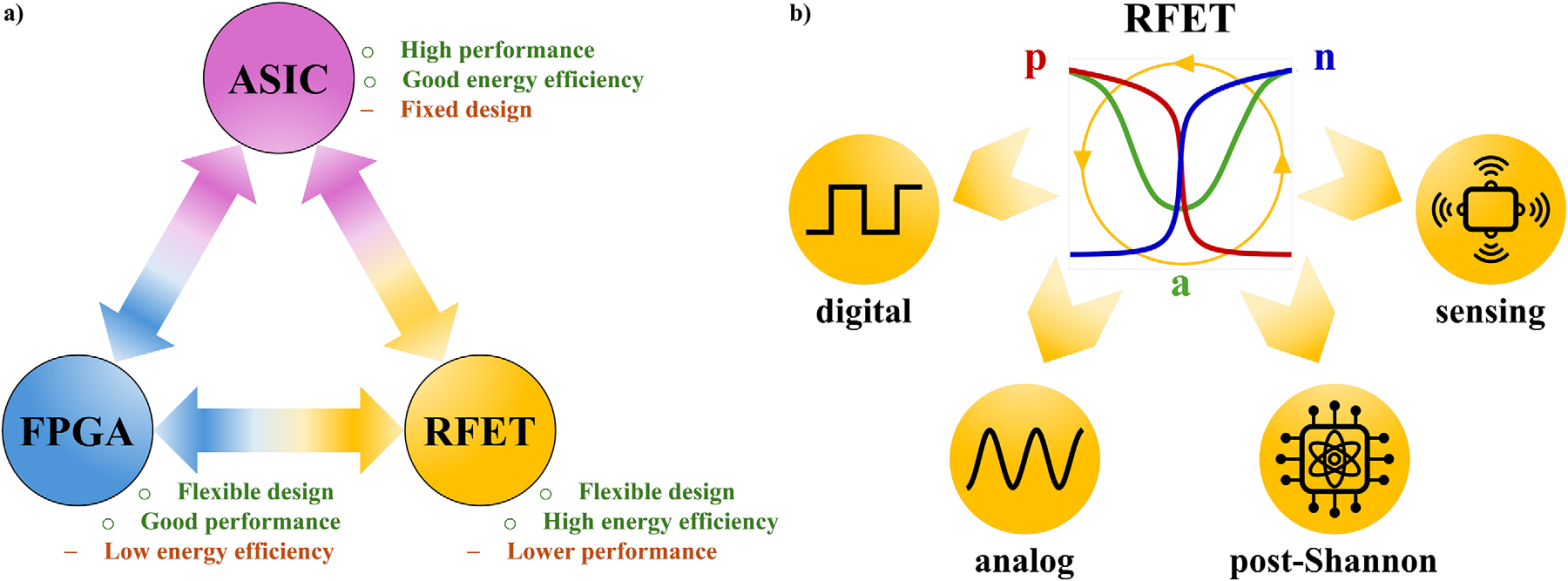
Overview of the reconfigurable field effect transistor (RFET) application space. (a) Positioning of RFET‐based fine‐grain reconfigurable architectures between fixed‐function application‐specific integrated circuits (ASICs) and coarse‐grain field‐programmable gate arrays (FPGAs). (b) Representative sketch of the adjustable functionality of RFETs, including *n*‐type, *p*‐type, and ambipolar mode, as well as the main areas of application discussed within this review.

Unlike other emerging device concepts, the benefit of employing RFETs is not directly visible in typical device‐level figure‐of‐merit metrics, such as on‐current or subthreshold slope [3]. Instead, circuit‐ and system‐level benefits are achieved by tuning the circuit application to exploit the underlying device properties as efficiently as possible. As a consequence, RFETs require designers to rethink their systems to leverage their flexibility to the maximum. If executed successfully, not only can complex functions be mapped into fewer elements, leading to simplified and more energy efficient circuits, but also an additional layer of adaptability can be added to the system, ultimately leading to more powerful, reusable and sustainable hardware solutions for a variety of application scenarios, including low‐power edge‐computing, neuromorphic computing, analog signal processing, bio‐sensing, as well as security and cryptography, as summarized in Figure 1b.

This paper provides a comprehensive review of the state of the art in RFET circuit design. It begins by introducing the physical principles underlying RFET operation and proposes a revised gate nomenclature to improve consistency across future RFET‐related work. It then discusses device implementations across a wide range of material systems, including Si, Ge, III–V semiconductors, carbon nanotubes, and two‐dimensional materials such as ${\mathrm{MoS}}_{2}$, black phosphorus, and graphene. Key achievements in digital, analog, and post‐Shannon circuit design are reviewed and exemplified on new measurements on an FDSOI technology. The paper concludes with an outlook on the possibilities and challenges in reliability, back‐end‐of‐line integration, and electronic design automation (EDA) needed for system‐scale‐up of future reusable and sustainable electronics.

## 2 RFETs and Their Properties

Reconfigurable field effect transistors (RFETs) are emerging electronic devices capable of conducting both holes and electrons without altering their physical device structure. Therefore, the same physical device can act as either an *n*‐type, *p*‐type, or ambipolar transistor. In conventional MOSFETs, the polarity is set during the fabrication process, forcing a device to rigidly behave as *n*‐type or *p*‐type. In contrast the polarity of reconfigurable transistors can be modified at runtime by applying different potentials (i.e, electrostatic doping). This feature significantly differentiates RFETs from conventional MOSFETs, ultimately enabling their exclusive properties.

While some RFET variants employ band‐to‐band‐tunneling [4], impact ionization [5], or positive feedback mechanisms [6], most RFETs demonstrated in hardware are Schottky barrier‐based devices realized on undoped semiconductor nano‐channels and controlled by multiple independent gates [7]. As such, these devices are the focus of this work, although other variants are mentioned in application‐specific scenarios.

### 2.1 Device Variants and Naming Conventions

Most hardware‐realized RFET variants rely on the formation of Schottky barriers (SBs) between a semiconductor channel and metallic source and drain contacts. These SBs allow the device to conduct both electrons and holes, as both types of carriers can tunnel through them. Moreover, the devices typically utilize undoped channels, which facilitate symmetric operation between the *p*‐ and *n*‐type configurations. Together, these material properties provide the foundation for device polarity control by enabling ambipolar transport with comparable conduction of both carrier types. However, ambipolarity alone does not determine the device polarity. Only through the application of appropriate electrostatic potential combinations can the same device be configured to exhibit either *p*‐type or *n*‐type conduction [2, 7].

While some devices with a single gate structure [8, 9, 10] have been presented, most SB‐based RFETs require at least two independently addressable gates: one to set the device polarity (*polarity gate(s)*) and the other to steer the device (*control gate(s)*). The polarity control mechanism initially relies on biasing the device with a voltage applied between its source and drain terminals, followed by identifying the carrier‐injecting barriers for both polarities, and finally on individually gating those barriers. The bias polarity defines the direction of carrier transport and, consequently, which contact acts as the carrier‐injecting terminal for electrons or holes. By independently modulating the electrostatic potential at the corresponding injection barrier, the barrier can be thinned to promote tunneling of the desired carrier type or widened to suppress its injection. In this way, appropriate gate‐bias combinations selectively enable electron or hole conduction, allowing the same device to operate in either the *n*‐type or *p*‐type configuration.

The gates of SB‐based RFETs can be described according to two fundamentally different criteria: their physical position within the device or the function they perform under a particular biasing scheme. A positional nomenclature is independent of the operating mode and can therefore be applied consistently across different RFET architectures. Gates located above the semiconductor channel are collectively referred to as *top gates* [11], whereas *bottom gates* lie below the semiconductor channel. Along the transport direction, localized gates overlapping the source‐ and drain‐side Schottky junctions are denoted as the *source gate* (SG) and *drain gate* (DG), respectively, as illustrated for the dual‐independent‐gate RFET (DIG‐RFET) in Figure 2a. A localized gate positioned at the central channel region is referred to as the *central gate* (CG) [7]. Such an arrangement is found, for example, in three‐independent‐gate RFETs (TIG‐RFETs), in which the SG, CG, and DG form three independently addressable top gates, as shown in Figure 2b. Importantly, these labels describe the physical position of a gate and are independent of the total number of gates in the device; accordingly, RFET architectures containing only a single central gate have also been demonstrated, as illustrated in Figure 2c. In contrast, other architectures propose a single wider gate extending over most or all of the channel from above; such a gate is referred to as a *front gate* (FG). Conversely, a wide gate located beneath the semiconductor (a bottom gate) and electrostatically coupled to most or all of the channel is referred to as a *back gate* (BG). An RFET architecture incorporating both an FG and a BG is shown in Figure 2d. In gate‐all‐around (GAA) structures, the distinction between top and bottom gates is no longer meaningful. When independently addressable gated sections are distributed along the transport direction, they can instead be identified according to their longitudinal position, for example, as SG, CG, and DG.

**FIGURE 2:**
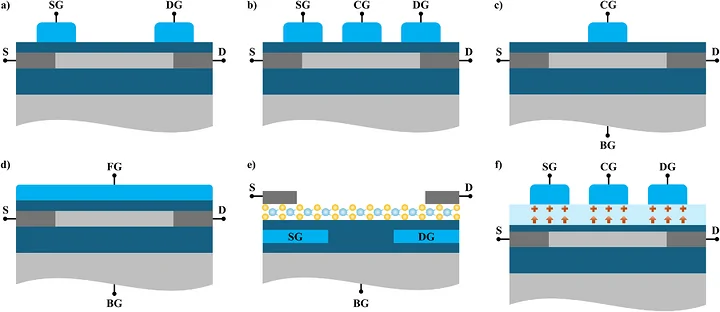
Schematic cross section of different RFET vairants. (a) Dual‐independant‐gate (DIG)‐RFET, (b) Three‐independent‐gate (TIG)‐RFET, (c) RFET with center‐ and back‐gate control (d) RFET with front‐ and back‐gate covering both Schottky barriers, (e) 2D‐RFET with source and drain contact above the channel and gate aligned to the contacts, and (f) non‐volatile TIG‐RFET with ferroelectric insulator or charge trapping stack above the channel.

In contrast, functional nomenclature describes the role assigned to a gate under a particular operating condition. This distinction can be illustrated by considering the *n*‐type operation of an RFET with gates located at the Schottky junctions, such as the devices shown in Figure 2a,b. In the *n*‐type configuration, electrons tunnel from the source‐side junction into the conduction band and propagate toward the positively biased drain, while the reverse‐direction injection of holes is suppressed at the drain‐side junction. The complementary *p*‐type configuration follows the same general principle, with the carrier types and corresponding electrostatic potentials reversed. Under these conditions, the gate controlling the drain‐side junction primarily determines the device polarity by suppressing injection of the undesired carrier type, whereas the gate controlling the source‐side junction primarily enables injection of the desired carriers and modulates the current through the device. In the literature, these two functional roles have frequently been described as *programming* and *control*, with the corresponding gates termed the *program gate* and *control gate*, respectively. Other works similarly employ terms, such as *polarity gate* for the gate responsible for selecting the active carrier type and *steering* for the control function. Importantly, these terms describe the role performed by a gate under a specific biasing scheme rather than the identity of a fixed physical terminal.

The coexistence of positional and functional naming conventions has resulted in a broad and often inconsistent terminology throughout the RFET literature. The same physical gate can be assigned different names depending on the selected operating scheme, while identical terms or abbreviations can refer to distinct physical or functional concepts. A particularly evident example is “CG”, which may denote either a *central gate* in a positional sense or a *control gate* in a functional sense. In addition, terms such as *top gate*, *front gate*, *back gate*, *bottom gate*, *middle gate*, *buried gate*, and *junction gate* have been employed according to different geometrical conventions, in some cases with partially overlapping meanings. The term *programming* introduces an additional ambiguity when RFETs are combined with non‐volatile memory concepts, where programming already denotes a distinct physical operation. Such inconsistencies become increasingly problematic as RFET research extends from device physics toward circuit design and system‐level applications, bringing together research groups with different areas of expertise and traditionally different terminology.

Naturally, there have been various attempts at classification across all types of RFETs. Besides classification based on the physical arrangement of the gates, described above, classification by transport mechanisms, or the employed channel material, are most often used in the literature. However, because some variants, such as the TIG‐RFET, can support multiple operation modes, these former metric often yields a confusingly high number of apparently similar device versions.

To reduce this ambiguity, in this work, we propose to explicitly separate the physical identity of a gate from its functional role. Physical gates are named according to their location and geometrical extent. Localized gates are identified along the transport direction as *source gate* (SG), *central gate* (CG), or *drain gate* (DG). These gates may be implemented either above or below the semiconductor channel and can therefore be further described, when necessary, as *top* or *bottom* gates. The term *front gate* (FG) is reserved for a wider top gate encompassing most or all of the channel, while the term *back gate* (BG) is correspondingly reserved for a wider bottom gate located beneath the semiconductor and encompassing most or all of the channel. For GAA structures, where a top–bottom distinction is not applicable, localized gates are identified solely according to their position along the transport direction.

The operating function of these physical gates is described independently. A physical gate, or combination of gates, whose primary role is to configure the carrier polarity is referred to functionally as a *polarity gate*, with the corresponding applied bias denoted by the *polarity voltage* ($V_{P}$). For the modulation of carrier transport, we adopt the term *control*: the corresponding physical gate, or combination of gates, is referred to as a *control gate*, and the applied bias is denoted by the *control voltage* ($V_{c}$). Depending on the device architecture and selected operating mode, the polarity and control functions may therefore be assigned to the SG, CG, DG, FG, BG, or to a combination of physical gates. Finally, a gate, or combination of gates, used to modify a non‐volatile state is referred to, functionally, as a *programming gate*. The corresponding biases are denoted by the *programming voltage* ($V_{\mathrm{PRG}}$) and, where applicable, the *erase voltage* ($V_{\mathrm{ERS}}$), following conventional terminology used in the memory community.

The functional terms *polarity gate*, *control gate*, and *programming gate* are deliberately not abbreviated, thereby maintaining an unambiguous distinction between the physical identity of a gate and the role it performs. In device‐centric discussions, we encourage the use of positional nomenclature and therefore refer directly to terminal voltages such as $V_{\mathrm{SG}}$ or $V_{\mathrm{CG}}$. In circuit‐ and system‐level discussions, where the functional role is typically more relevant than the physical gate position, we instead suggest focusing on the applied signals or physical quantities such as the voltages $V_{P}$, $V_{c}$, $V_{\mathrm{PRG}}$, and $V_{\mathrm{ERS}}$, independent from their exact physical terminal.

### 2.2 From Nanowires to Industrial FDSOI

Over the years, a variety of RFET variants and application scenarios have been described. The main milestones discussed throughout the paper are highlighted in the timeline in Figure 3. The origin of the technology dates back to the year 2000, when researchers investigated a solution to suppress the undesired ambipolar branch in Schottky‐barrier‐based thin‐film transistors [12, 13]. They found a solution with two independent gate electrodes, in which the control gate overlapped the source contact (i.e., SG), whereas the DG, then called field‐induced drain extension, was employed to suppress the undesired carrier type (i.e., polarity‐gate), yielding unipolar behavior. The concept was rediscovered 8 years later in industrial research on transport in nanowires with silicided source and drain contacts [14].

**FIGURE 3:**
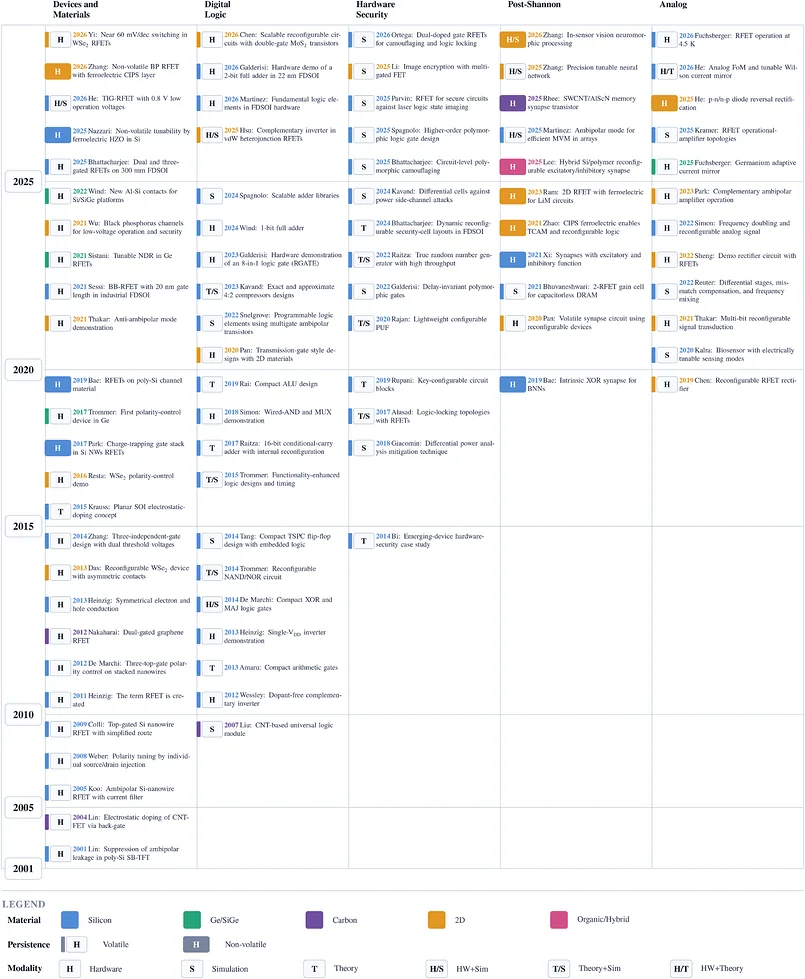
Timeline on selected RFET development milestones.

The term reconfigurable field‐effect transistor (RFET) was coined only a few years later in 2011 [1], demonstrating a more advanced version of the dual‐independent‐gate (DIG‐RFET) approach and highlighting the potential dynamic reconfiguration in CMOS designs for the first time. Two years later, the first dynamic reconfigurable inverter with equal electron and hole transport was shown using the same device layout, marking the first true complementary circuit demonstration, allowing for a cascading of logic functions [15].

Around the same time, an alternative approach to polarity‐reconfigurable transistors was demonstrated in carbon nanotube FETs employing a local central gate in combination with a global back gate [16]. The back‐gate voltage controls carrier injection at the Schottky contacts and electrostatically dopes the adjacent CNT regions, forming either a (p/i/p) or (n/i/n) profile, while the central gate controls carrier transport through the intrinsic channel region (Figure 2c). Reversing the back‐gate polarity, therefore, selects between *p*‐type and *n*‐type operation. Already by 2007, dynamically reconfigurable CNTFET‐based look‐up table (LUT) like circuits had already been proposed theoretically, making a first digital circuit design example for RFETs [17].

Interestingly, this CNT architecture with CG and BG can be regarded as an early predecessor of the TIG‐RFET concept highlighted above, as it separates polarity control at the Schottky barriers from transistor switching in the center of the channel. Subsequent implementations replaced the global back‐gate control with local gates acting near the source and drain junctions, allowing the required polarity‐control voltages to be reduced to logic‐compatible levels, and thereby facilitating the cascading of transistor stages. This was demonstrated in silicon nanowire technology in 2012 using a central control gate and a polarity gate acting on both source‐ and drain‐side regions [18], and was later extended to three independently controlled gates in the TIG‐RFET concept [19]. Other early works heavily adopted silicon nanowires, showcasing various strategies to achieve unipolarity from the underlying ambipolar baseline technology [20, 21, 22].

In the following years, research focused on device demonstrators utilizing top‐down fabrication methods [23]. Among other variants, also RFETs with a planar SOI channel geometry were predicted [24] and first demonstrated in 2018 [25]. Despite the wider nanosheet channel, the cross‐section design remained very similar to the earlier nanowire‐based iterations. Still, the design gave rise to a transfer industrial implementation with considerably less process adjustments as compared to CNTs or Si nanowire technologies. In 2021, a completely different type of reconfiguration was first demonstrated in industrial SOI hardware. Bringing the metal leads at the source and drain closer together results in a design where both front‐ and back‐gate couple to both Schottky junctions simultaneously [26], as seen schematically in Figure 2d. This concept, called *back bias RFET* (BB‐RFET), operates on a notably different principle. While DIG‐ or TIG‐RFET variants can achieve unipolar operation by independently steering each Schottky Barrier, the ambipolar base operation in the BB‐RFET is well preserved across all program voltages. Instead of blocking the undesired carrier, only the minimum operating point is shifted along the control‐gate voltage axis, leading to interesting features in the analog signal‐processing domain [27]. Finally, in 2025, the TIG‐RFET discussed above was also transferred to industrial FDSOI hardware [28], combining the highly versatile design opportunities of TIG‐RFETs with feature‐independent back‐gate control [29]. A schematic of the device is shown in Figure 2a. In 2026, even a device variant requiring as little as 0.8 V across all terminals was demonstrated, highlighting the potential of RFETs for low‐power circuit design [30].

In parallel to the progress in silicon‐based devices, reconfigurable transistors employing layered two‐dimensional (2D) channel materials have also been experimentally demonstrated, including graphene [31, 32], ${\mathrm{WSe}}_{2}$ [33, 34, 35], and ${\mathrm{MoTe}}_{2}$ [36, 37]. In principle, 2D‐RFETs share the same structural features as silicon‐based devices but often require an additional contact‐gating strategy due to the absence of ultra‐sharp diffusive metalides. Thus, typically bottom gate concepts (sometimes called buried gates in the 2D community), placed directly below the source and drain top‐side contacts, are used to enhance carrier injection (Figure 2e). Among them, in particular black phosphorus has emerged as an interesting channel material for RFETs, yielding reasonably high performance at low operating voltage [38, 39]. Ambipolar conduction and reconfigurable circuits have also been demonstrated on organic platforms [40, 41]. Hybrid material systems have likewise been explored, including silicon nanowire RFETs incorporating 2D hBN [42], as well as devices combining 2D materials with functional organic layers [43, 44]. Interestingly, all Schottky FET‐based variants are largely independent of the underlying channel material, as long as a good gate control of carrier injection at a near‐midgap Schottky barrier can be achieved [7]. In addition, low‐bandgap materials are often more prominent due to their higher theoretical performance. A comprehensive overview of the state of the art in 2D‐based RFETs is provided in Section 2.4. Design solutions, such as incorporating a charge trapping [45] ferroelectric layer [46] to achieve non‐volatile performance, are also possible (Figure 2f). This approach holds great promise, particularly to store the polarity state without the need for a continuous supply voltage. RFETs with non‐volatility are discussed in detail in section 4. For a more comprehensive historical overview of Schottky–Barrier‐based RFETs, readers are referred to [7].

### 2.3 The Many Faces of RFET Operation

Figure 4 illustrates the potential for adaptive operation of a TIG‐RFET based on a device realized in 22nm FDSOI technology [28]. The device layout is illustrated in panel 4a. By using electrostatic control, a single RFET achieves both *p*‐type and *n*‐type (Figure 4b) conduction. Figure 4c,d shows the *n*‐ and *p*‐type biasing configurations when the transistor is controlled at the central gate (CG control), with the polarity voltage ($V_{P}$) applied as bias to SG and DG. The transfer curves show excellent symmetry, with both branches reaching a strong on/off ratio larger than $10^{5}$. In the subthreshold region, the curves overlap perfectly even when varying the drain‐source potential ($V_{\mathrm{DS}}$), thus the threshold voltage ($V_{\mathrm{th}}$) is constant, indicating highly stable electrostatic control. Sweeping all three front gates simultaneously produces ambipolar behavior (Figure 4e) because of the Schottky barrier FET nature of RFETs. Conversely, sweeping SG and DG together in one direction, and the inner gate in the opposite direction, yields the anti‐ambipolar profile seen in Figure 4f. Both ambipolar modes naturally facilitate applications, such as frequency doubling [27, 47], whereas the selective anti‐ambipolar behavior can be used as an analog comparator or window detector.

**FIGURE 4:**
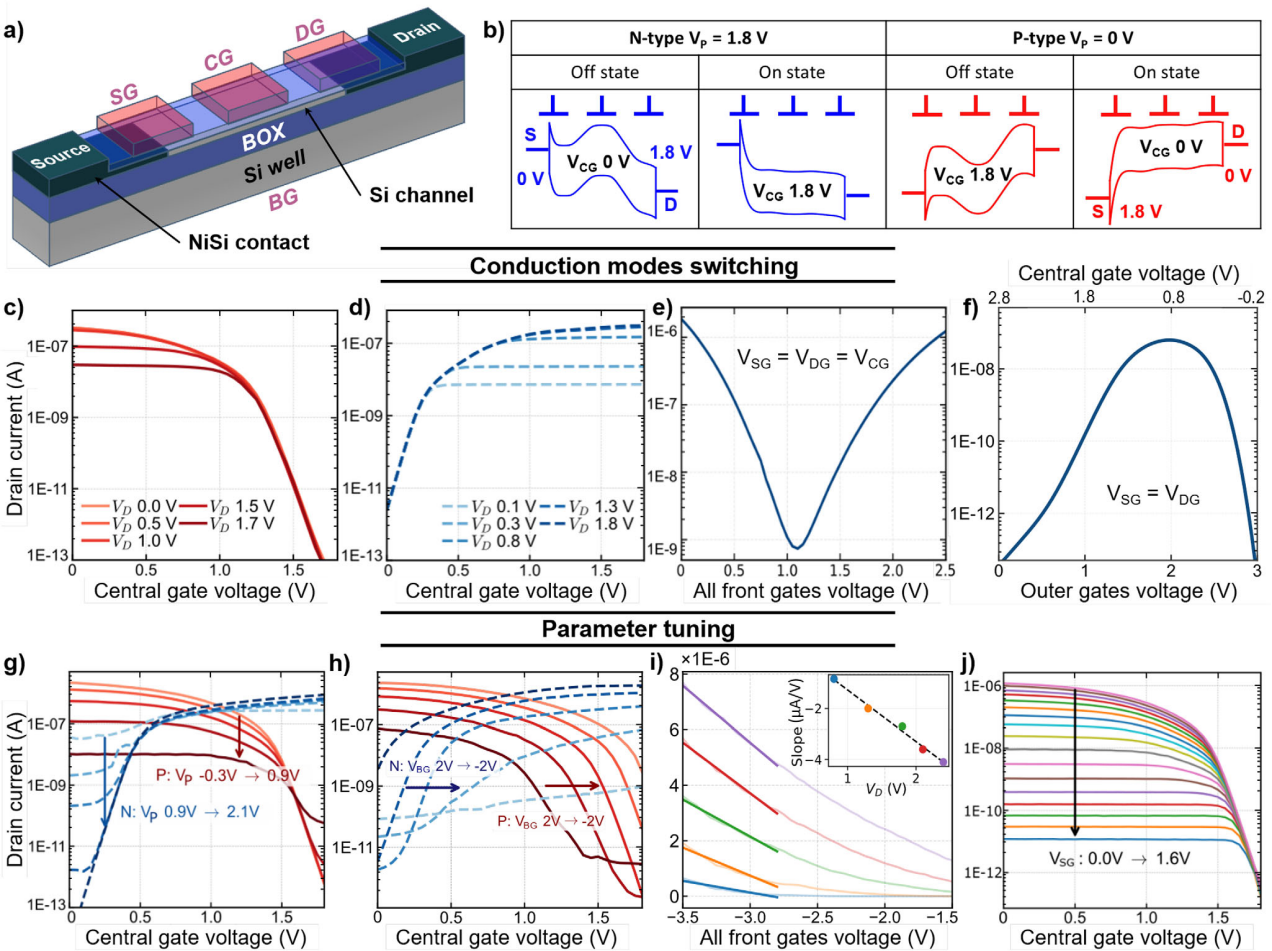
Three‐Independent‐Gate (TIG)‐RFET operation regimes. (a) 3D schematic of the TIG‐RFET in 22nm FDSOI technology. (b) Band diagrams of TIG‐RFET in CG steering mode, illustrating the on and off states under n‐ and p‐type bias conditions, respectively. (c) and (d) *p*‐ and *n*‐type transfer characteristics under varying drain voltages V_D_. (e) Ambipolar transfer characteristics with linear fitting, highlighting the linear operating regions. (f) Anti‐ambipolar characteristics. (g) Transfer curves under varying V_P_. (h) Transfer curves under varying V_B_. (i) Linear scale plot of the I_D_‐V_G_ for the *p*‐type branch under varying positive V_D_. Linear fits with $R^{2}>0.98$ are shown in a thicker line. The inset shows the fitted slope as a function of the drain voltage, which also shows a linear trend (fitted dashed line). (j) *P*‐type transfer curves under varying V_SG_, showing injection limited transfer behavior.

Beyond these four conduction modes, the extra degree of freedom provided by the multi‐gate structure enables parameter tuning. Figure 4g demonstrates that adjusting $V_{P}$ (simultaneously at SG and DG) provides control over both the on and off states of the device. The source‐gate potential $V_{\mathrm{SG}}$ limits carrier injection at the source side (governing the on‐state), while the drain‐gate potential $V_{\mathrm{DG}}$ controls the blocking of opposite‐type carriers at the drain side (governing the off‐state). Additionally, applying a back‐bias shifts both the *p*‐ and *n*‐transfer curves concurrently in the same direction (Figure 4h). For example, a positive back‐bias bends the energy bands downward, thinning the barrier and making conduction more favorable for electrons. Notably, if used in ambipolar operation with higher gate voltages, the device enters a linear transfer regime due to velocity saturation [48]. For a positive $V_{\mathrm{DS}}$, the left branch at negative gate potentials corresponds to hole‐injection from the drain side. The slope of this linear region scales linearly with $V_{\mathrm{DS}}$, shown on a linear scale in Figure 4i. This bi‐linearity is highly advantageous for in‐hardware computation, where linear weight tuning is critical for matrix multiplication.

Moreover, Figure 4j illustrates injection‐limited behavior under multiple $V_{\mathrm{SG}}$ values and at fixed $V_{\mathrm{DG}}=0\;V$. Because the source gate restricts the amount of carriers injected through the Schottky barrier [49, 50], the transfer curve eventually saturates, resulting in near‐zero transconductance. This saturation is ideal for certain applications, such as matrix pixel drivers [51]. In these displays, the circuit must supply a distinct current level to control specific pixel intensities. Once the device reaches this threshold, the flat transconductance plateau ensures that the driving current remains stable and immune to minor voltage fluctuations [52, 53].

### 2.4 Low Bandgap Materials

The selection of the semiconductor material is pivotal for optimizing RFET performance, with low‐bandgap channel materials playing a particularly important role. Materials with smaller band gaps, such as silicon‐germanium and germanium [11, 29, 54], or selected III–V compound semiconductors [55, 56, 57], can enhance carrier injection through the Schottky junctions by reducing the barriers for both electrons and holes and by providing lower tunneling effective masses. This results in increased tunneling currents and improved ON‐state performance, which is particularly relevant for RFETs, where current transport is often injection‐limited. The use of multiple independently controlled gates can, at the same time, mitigate the increased source–drain leakage typically associated with low‐bandgap channels by suppressing the undesired carrier type directly at the corresponding injection barrier [54], thereby reducing static power consumption. Lower injection barriers can also reduce the voltages required for device operation, since a smaller electrostatic band shift at the junctions is needed to reach the ON‐state. Consequently, low‐bandgap RFETs can achieve higher drive currents at lower gate and supply voltages than comparable Si‐channel devices, thereby reducing the dynamic power consumption of digital circuits. In addition, the higher carrier mobilities commonly available in low‐bandgap semiconductors can further improve transconductance and switching speed.

Another key aspect is the use of metal work function engineering as a central strategy for controlling carrier injection in RFETs. The height and polarity of these barriers, and thus whether electrons or holes are preferentially injected, are largely determined by the metal's work function relative to the semiconductor electron affinity. High–work function metals such as Ni (~5.1 eV), Pd (~5.4 eV) or Pt (~5.6 eV) tend to align closer to the valence band of silicon after considering the Fermi‐level pinning conditions, facilitating hole injection and favoring p‐type operation [23, 58, 59]. In contrast, low work function metals like Al (~4.2 eV) or Ti (~4.3 eV) ideally align closer to the conduction band, promoting electron injection and enabling n‐type behavior [60, 61]. By selecting appropriate contact metals, or even combining them asymmetrically, RFETs can be tuned for enhanced ambipolar performance or biased toward one carrier type. In terms of inorganic channels, especially the diffusive formation of metallic alloys, such as ${\mathrm{NiSi}}_{2}$ [62], but also single elemental metals [61], by thermally assisted solid‐state reactions, is widely used to form abrupt and flat Schottky junctions. Thereby, the metal segment is intruded into a semiconductor nanochannel via thermal annealing as a single‐crystal material, enabling the creation of atomically sharp and flat interfaces. Such junctions lead to confinement of the contact area to the channel cross‐section and superior gate control over the injection due to electrostatic field enhancement at the edges. A variety of metal‐semiconductor combinations have been reported, with the well‐known silicides NiSi‐Si and ${\mathrm{NiSi}}_{2}$‐Si, and the germanides NiGe‐Ge, being the most prominent. Nevertheless, most studies show a slightly lower barrier height for hole injection than for electron injection using those metal compounds. More recently, single‐elementary Al has emerged as a contact material for nanometer‐scale channels of Ge, Si, and SiGe, Figure 5a–d). Different to silicide formation, this process is driven by exchange reaction, where the Si and/or Ge atoms outdiffuse and are replaced by Al atoms, given the high diffusion constants of Ge and Si atoms in Al. As a result, single‐crystal Al leads are formed, making an abrupt junction to Si and to Ge channels (Figure 5b–d). For pure Ge channels, Fermi‐level pinning is close to the valence band, promoting strong p‐transport, as demonstrated in Ge nanowires and on insulator substrates [63]. In the case of Si, this is significantly different, the Fermi‐level pinning is close to the midgap, making it an interesting contact for symmetric RFET operability. For SiGe and Ge on SOI channels, the exchange reaction involves both Si and Ge, which turns into the formation of a reproducible hetero‐interlayer at the junctions. The Ge or SiGe channel is followed by a 3–5nm thin single‐crystal epitaxial Si interlayer and a single‐crystal Al layer that re‐establishes Fermi‐level pinning near the middle of the Si band gap (Figure 5b–d). However, because the Si barrier is thinner than in a full Si device, tunneling injection is far more efficient. Thereto, SiGe and Ge RFETs with a high level of symmetry and on currents have been demonstrated (Figure 5e). Different from polycrystalline Al films employed in the early phases of MOSFETs, there is no visible degradation of the Al segment and junctions upon extended operation. Nevertheless, a systematic reliability study at higher temperatures is pending. The pure Al junction also offers benefits for cryogenic electronics [64], as Al becomes superconducting at cryogenic temperatures [65]. Al–Ge–Al heterostructures have enabled the realization of gate‐tunable single‐hole transistors [66] and Josephson FETs exhibiting proximity‐induced supercurrents through intrinsic Ge [67].

**FIGURE 5:**
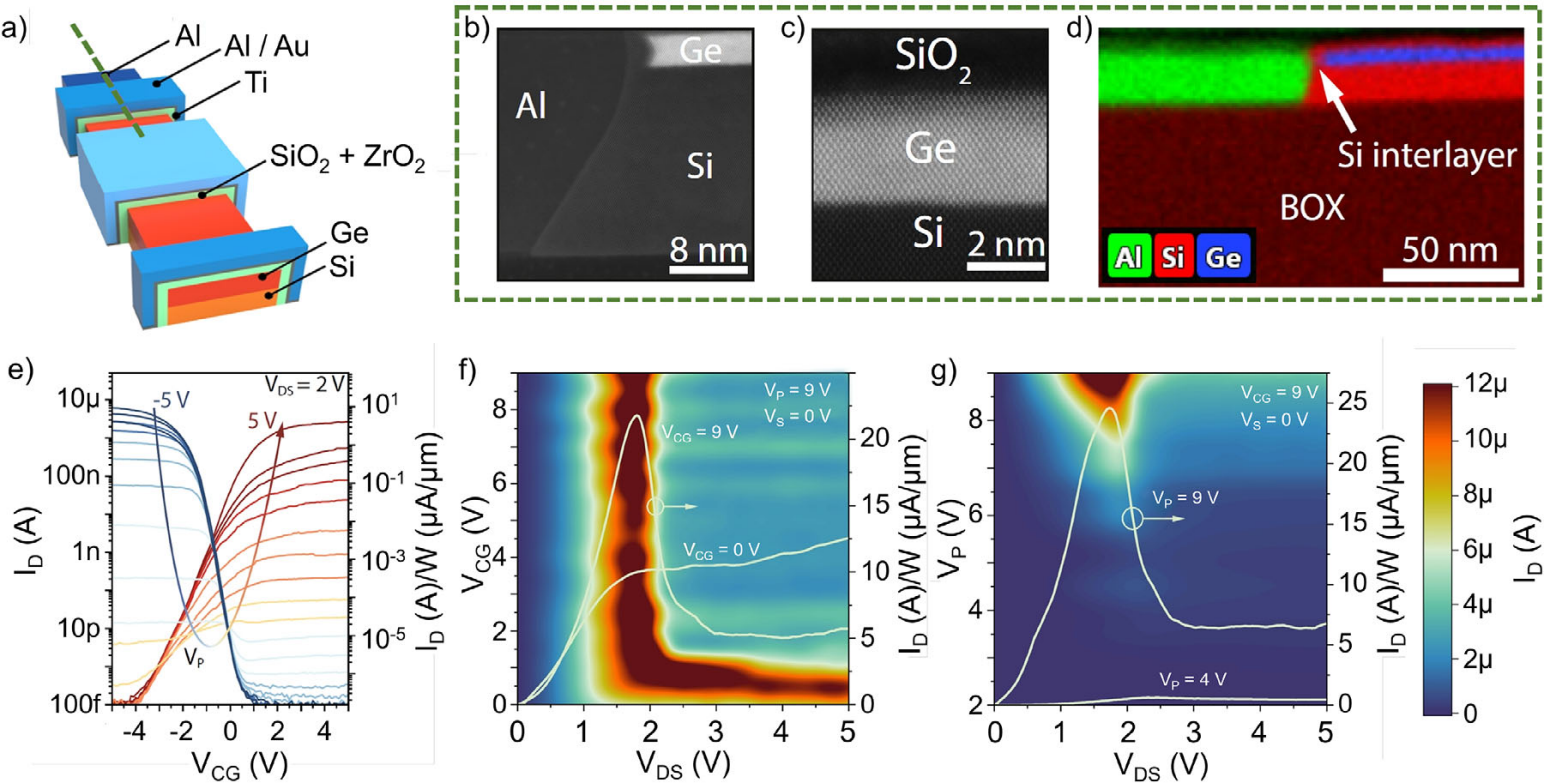
Ge on SOI RFET with Al contacts. (a) Schematic view of the device material composition and architecture, showing the cross‐section line for TEM in (b) – (d). TEM cross‐sectional views across the junction showing b) exchanged Al within the channel, delivering a sharp interface between Al and the semiconductor, and (c) the epitaxial quality of Ge on SOI layer. (d) Elemental mapping with EDX showing Al, Si, and Ge composition. (e) Subthreshold transfer characteristics with high level of symmetry given by the Si interlayer between Ge and Al, ${\mathrm{ZrO}}_{2}$ below the gate centers the subthreshold characteristics at $V_{\mathrm{CG}}$ toward 0 V. (f) Setting the polarity of the device as n‐FET $V_{P}$ = 9 V, the output characteristics are recorded for multiple $V_{\mathrm{CG}}$. The device can provide conventional $I_{\mathrm{DS}}$ saturation or NDR depending on $V_{\mathrm{CG}}$, whereas (g) $V_{P}$ can tune the NDR peak current.

Another interesting aspect of employing Ge channels with Al source/drain contacts is the observation of negative differential resistance (NDR) at room temperature in the output characteristics of the devices. This already holds for the single‐gated Schottky barrier FET [68] as well as for RFET designs with two (DIG‐RFET) [69] or more gated regions (TIG‐RFET), see Figure5f. The observed NDR effect only occurs for pure Ge channels and for *n*‐type operation of the devices [70]. The effect is attributed to the Hillsum–Gunn transferred‐electron effect, where accelerated electrons scatter from the low‐effective‐mass L valley to the X valley with higher effective mass, thereby decreasing channel conductance [63]. By integrating Ge on SOI into a TIG‐RFET structure, it is possible to incorporate a switchable, tunable NDR effect into the polarity‐control functionality [70]. When configuring the TIG‐RFET with NDR feature to n‐conduction, the polarity voltage applied at both junctions (DG and SG) can exponentially tune the height of the NDR peak current, see Figure 5g. Moreover, the control voltage can be used to switch the NDR effect on and off with a drain voltage of only 1.8 V, as it modulates the electric field across the channel; see Figure 5f. First circuit implementations have been demonstrated using Ge‐based NDR‐RFETs, like a cascoded circuits of two NDR‐RFETs that generate double NDR features in the output characteristic [71].

### 2.5 Two‐Dimensional Materials

The operation of RFETs strongly depends on the electrical and structural properties of the semiconductor, since both the selective injection and the efficient modulation of electrons and holes are governed exclusively by external electric fields. In this context, low‐dimensional materials are theoretically advantageous over three‐dimensional (3D) bulk semiconductors [72]. Owing to their atomically thin, continuous, and dangling‐bond‐free structure, 2D materials enable excellent electrostatic gate coupling while avoiding the severe mobility degradation observed in bulk semiconductors at channel thicknesses below approximately 5 nm [73]. In particular, the required presence of Schottky barriers in RFETs makes efficient tunnel injection at the contacts essential for achieving high on‐currents for *p*‐ and *n*‐configuration. The large variety of available 2D semiconductors, together with the partially thickness‐dependent bandgap, theoretically enables the threshold voltages of both RFET polarities to be tuned over a wide range. The intrinsically closed and dangling‐bond‐free surface structure of 2D materials offers unprecedented freedom to combine different 2D materials into so‐called van der Waals heterostructures without the stringent lattice‐matching requirements commonly encountered in bulk materials [74]. The most prominent representatives of 2D materials include graphene as a semimetal, transition metal dichalcogenides (TMDs) as semiconductors, and hexagonal boron nitride (hBN) as an insulator. As described by the operating principle, the active materials employed in RFETs must support both electron and hole injection and conduction. Ambipolar 2D semiconductors such as ${\mathrm{WSe}}_{2}$ [33, 34, 35], ${\mathrm{MoTe}}_{2}$ [36, 37], and black phosphorus [38, 39, 75], therefore constitute a fundamental prerequisite for the realization of 2D RFETs [76]. Although a wide range of 2D materials exhibit nominal band alignments with commonly used contact metals such as Ti, Al, Cr, and Ag that would theoretically result in low injection barriers for both electrons and holes, only a limited number of experimentally realized contacts demonstrate truly ambipolar transport behavior [77]. One of the primary reasons for this discrepancy is the occurrence of Fermi‐level pinning at the metal/semiconductor interface. This effect is widely attributed to metal‐induced gap states (MIGS), which introduce interface states within the semiconductor bandgap and thereby constrain the energetic position of the Fermi level largely independently of the metal work function [78].

Based on ambipolar 2D materials, various RFET architectures have been demonstrated through electrostatic control of the Schottky junctions [39, 72, 79, 80]. For example, a ${\mathrm{WSe}}_{2}$‐based TIG‐RFET employing two bottom polarity gates below the source and drain contacts and a control gate in the middle of the channel for tuning carrier transport was demonstrated with ON/OFF ratios of $10^{7}$ for n‐type operation and $10^{6}$ for *p*‐type operation [34]. Pan et al. demonstrated, using independent Schottky gates on a ${\mathrm{WSe}}_{2}$‐based device, polarity control, reconfigurable logic circuits, and synaptic behavior [81]. Symmetric transfer characteristics, the prerequisite for CMOS‐integration, could be achieved by a ${\mathrm{WSe}}_{2}$/${\mathrm{MoS}}_{2}$ van der Waals heterojunction [82]. The best performing 2D RFET so far was based on black phosphorus (BP), achieving transistor‐level reconfigurability at sub‐1 V operating voltages, as well as NAND/NOR obfuscation for hardware security circuits [38]. While most of these studies are based on exfoliated multilayer 2D materials, several groups have synthesized large‐area 2D materials by chemical vapor deposition (CVD). For instance, a single‐layer CVD‐${\mathrm{WSe}}_{2}$ partially doped dual‐channel RFET demonstrated reconfigurability, non‐volatile programming, and a 2T reconfigurable Boolean logic functionality [83].

Despite the apparent theoretical advantages of 2D materials, no 2D‐based RFET has yet been demonstrated that outperforms devices based on conventional bulk semiconductors. This holds across key metrics relevant to RFET‐based electronics, including electrical symmetry, on‐current, subthreshold swing, and device size. Figure 6 illustrates this trend by comparing current density as a function of the highest applied gate voltage for selected RFETs reported in the literature. In general, RFETs based on 2D materials exhibit lower current densities and require higher operating voltages than their bulk‐semiconductor counterparts. Notable exceptions are two device demonstrators reported on BP channels [38, 39], whose performance was comparable to that of RFETs based on group IV semiconductors at applied voltages around 2 V.

**FIGURE 6:**
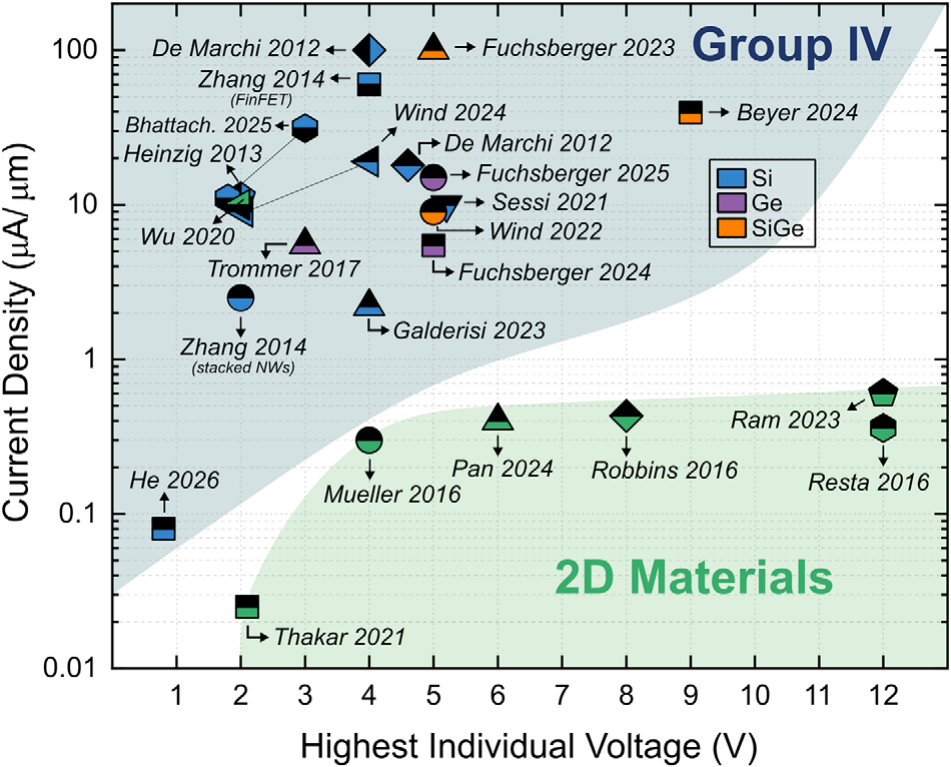
Benchmark of different RFET implementations with group‐IV semiconductor channels and 2D‐based channels.

The interfaces between the contact metals and 2D materials pose a major challenge, limiting the performance of the realized devices relative to their theoretical limits. On the one hand, Fermi‐level pinning typically results in increased barriers for hole injection, as reflected in, for example, the predominantly *n*‐type behavior of ${\mathrm{MoS}}_{2}$‐based transistors. However, weak orbital coupling, small effective contact area, and van der Waals gaps at the contact metals lead to increased contact resistance. Additionally, the van der Waals gap reduces electrostatic coupling of the gates to the channel due to the presence of an interfacial vacuum‐like gap in series with the gate insulator, effectively increasing the equivalent oxide thickness (EOT) [84]. Consequently, the low permittivity of the van der Waals gap weakens the electrostatic coupling between the gate field and the device, thereby negatively affecting device performance and scalability. To improve this electrostatic control, a recent ${\mathrm{WSe}}_{2}$ RFET incorporated capacitive amplification through a charge inversion layer and a geometrically asymmetric floating‐gate structure [85]. This approach amplified the electrostatic action of the control gate on the channel, enabling subthreshold swing to approach the Boltzmann limit while retaining conventional polarity‐gate‐controlled reconfigurable operation. Optimized Fermi‐level‐pinning‐free contact concepts and improved gate‐coupling strategies have been proposed; however, their superiority has not yet been conclusively demonstrated through high‐performance REFT demonstrators at scaled nodes and low operation voltages.

Whether superior RFETs can be realized using 2D materials depends on achieving truly ambipolar behavior with balanced electron and hole transport, reducing Fermi‐level pinning effects through low contact resistances, maintaining sufficient gate coupling despite the van der Waals gap, and enabling wafer‐scale deposition of low‐defect 2D semiconductor materials.

## 3 Digital Circuit Design and Applications

### 3.1 Gate Level Design

As highlighted in the introduction, RFETs demonstrate their added value primarily at the circuit level. From a digital perspective, the multi‐gated structure of a single RFET exhibits an all‐or‐nothing behavior, conducting current only when all gate voltages are in the same logic state (either high or low). For the design of digital logic gates, this translates into two properties that create a higher compactness: (a) an intrinsic XOR relation between the control and polarity gates [18], and (b) a wired‐AND functionality between multiple control gates in a single device [86]. Recent designs with multiple source contacts on a shared common body can even provide the wired‐OR counterpart, i.e., a transistor that is on whenever one of its inputs allows for conduction [87]. By employing these features, a higher level of compactness for arithmetic logic gates can be achieved. Gate‐level circuit demonstrators have been fabricated on a variety of channel materials and processing technologies. Thereby, the simplest way to demonstrate a circuit function is to couple a network of transistors to a load resistor, thereby yielding a dynamic logic gate [88, 89, 90, 91, 92]. If the transistor and supply voltages are reconfigured, a variety of functions can be mapped in a small number of elements. However, for static designs, both pull‐up and pull‐down networks must be implemented with the same technology, since the underlying devices provide symmetric threshold voltages and on‐currents [15, 22, 82, 93]. Other works have suggested highly compact pass‐transistor logic gates [81, 88, 94], which always require an extra inverter to drive the circuit.

Exploiting the higher‐order functionality of TIG‐RFETs, only three transistors are needed for a basic 2NAND or 2NOR gate, instead of four, as shown in Figure 7a,b. The costly 2XOR and 2XNOR functions can even be mapped into four transistors instead of eight [18, 95], see Figure 7c,d. The benefit increases with the complexity of the logic gate, e.g., for higher‐order Minority, Majority, or AND‐OR‐Inverter (AOI) gates [96]. In addition, many RFET‐based logic gates exhibit polymorphism, meaning they transform into their self‐dual counterparts when all supply and polarity voltages are reversed [97]. The most typically explored polymorphic designs are NAND/NOR [82, 98] and XOR/XNOR [38, 82], while other self‐dual designs, such as AOI/OAI [96, 99], have also been demonstrated. Interestingly, some functions, such as inverter and 3XOR, are reconfiguration‐invariant, meaning they retain the same function even if all supply and polarity voltages are reversed. While less useful from a computation perspective, such functions can be used in hardware security, e.g., to add noise or randomness to a system or to mask its actual reconfiguration [100, 101]. Often, these devices can also be operated in a transmission‐gate mode, providing higher‐order functionalities such as 3XOR or 3MAJ, as shown in Figure 7e, where they are employed together to realize a compact 1‐bit full adder [102, 103]. An efficient inverting multiplexer (MUX) can be realized using TIG‐RFET is comprising only six transistors [96], compared to the 10 required in traditional CMOS, if in both cases the required inverter for the select signal are included. While the impact of transistor count depends on relative device sizes, the RFET‐based implementation offers technology‐agnostic benefits. Indeed, it has only one transistor between the power rails and the output node and is fully static, resulting in the same driving strength as an inverter gate in the same technology, thus achieving optimal fan‐out and speed [104]. Multiplexer gates with extended capabilities have been proposed, which could perform the function of a two‐stage multiplexer [105], albeit these designs require more than 3 independent gates, which, while physically feasible, result in overwhelming complexity.

**FIGURE 7:**
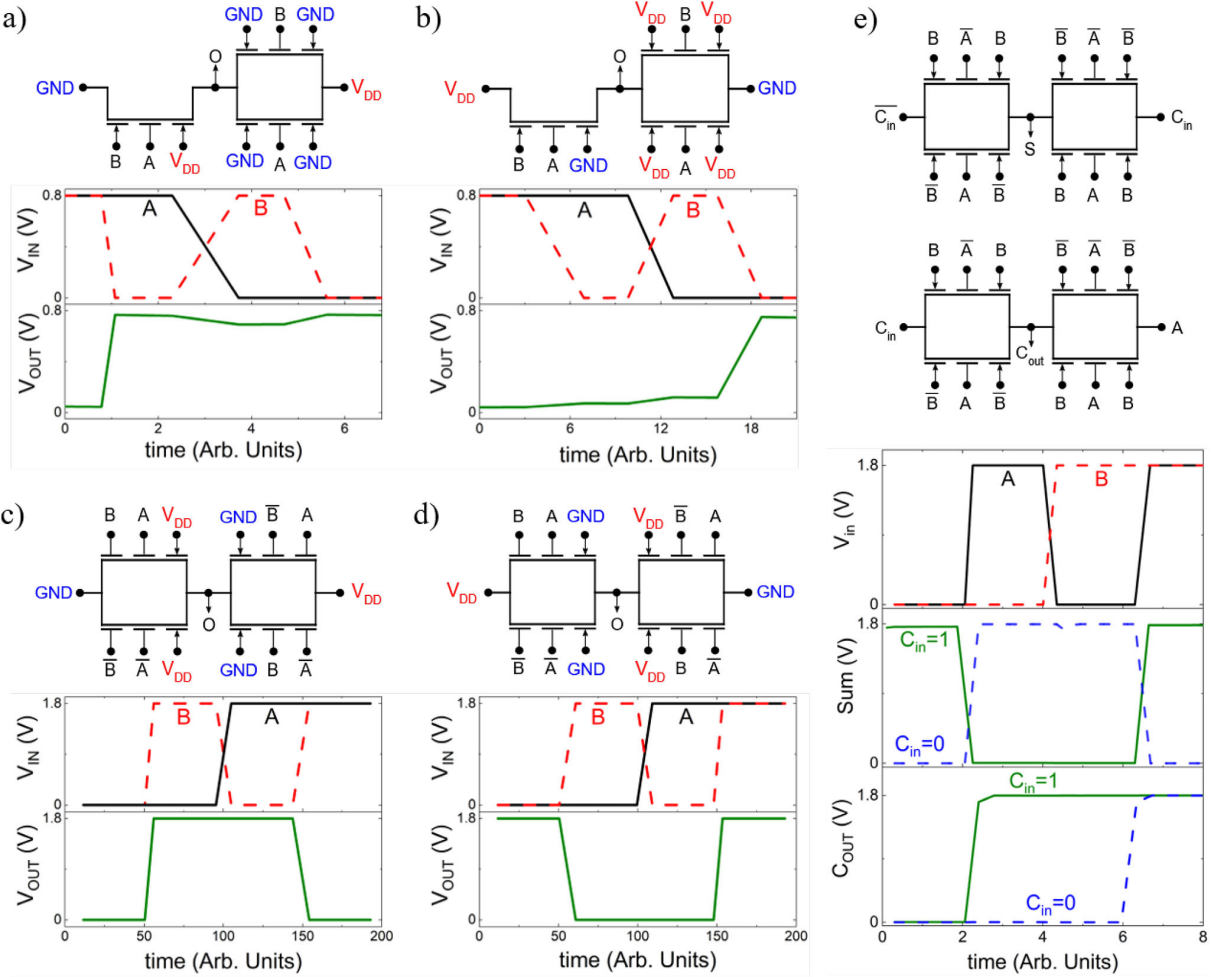
Basic digital RFET logic gates. (a) Electrical measurement of a NAND gate operating at $V_{\mathrm{DD}}=0.8\;V$, which can be reconfigured by switching $V_{\mathrm{DD}}$ and GND to yield the (b) NOR function. (c) Characterization of the XOR function, which can be reconfigured to realize (a d) XNOR gate. (e) Implementation of a three‐input XOR and MAJ gates, which together realize a 1‐bit full adder.

Recently, the first liberty and layout libraries have also been created for RFET‐based digital cells on 22nm FDSOI technology [99] and 14 nm FinFET technology [106]. However, they certainly do not comprise all possible functional variants for all those gates. Formal methods have shown that dynamic reconfiguration leads to a high variety of topologically different versions can be explored for RFET designs. Just for the simple NAND/NOR and 2XOR/2XNOR variants 18 and 49 distinct topologies could be derived, respectively, by formal methods, each with distinct power, performance, area (PPA), and symmetry trade‐offs [107, 108].

The reconfigurable concept has been well explored for static logic, but can also be applied to dynamic logic or pass‐transistor logic. Here, the unique multi‐gated structure can reduce charge‐sharing effects and increase the stability of logic styles, such as domino logic and dual‐rail logic [89, 109]. Beyond simple gate‐level designs, it has been shown that even more polymorphic functions can be achieved by combining multiple RFETs into universal logic modules (ULM). Already, early research on ambipolar carbon nanotube technologies has shown that multiple functions can be efficiently mapped, employing dynamic designs with stringent clock timing [17, 110, 111, 112]. Recently, compact static ULM designs have also been proposed [113]. One of the most compact designs, named RGATE, combines up to eight static functions with as few as four transistors solely through clever signal shuffling [114]. Similar designs have also been proposed by other groups [115]. In Ref. [116] a complex NAND‐XNOR‐XOR‐AND function was mapped to a gate with eight transistors, having nearly the same energy requirements as a simple XOR gate in the same technology.

Recent works have incorporated a non‐volatile storage function into reconfigurable devices, realizing compact implementations of reconfigurable gates capable of performing functions such as NAND, NOR, NOT and XOR [90, 117]. Crucially, in‐device storage eliminates the need for a constant bias voltage, potentially reducing power consumption. Other works have used reconfigurable Feedback FETs in a similar manner [118], demonstrating higher‐order functions such as a half‐adder [119]. By combining the intrinsic XOR functionality of the RFET with non‐volatile storage, it is possible to realize a content addressable memory (CAM) with only a single transistor per bit, as the XOR/XNOR matching operation can be realized directly at the device level [120]. These elements are promising for Computation‐in‐Memory (CIM) approaches, which are discussed in greater detail in Section 4.

### 3.2 Low‐Power Digital Circuits

Historically, the added value of RFETs in digital design was first explored for compact, low‐power arithmetic circuits. Here, the flexibility provided by the baseline ambipolar behavior of reconfigurable transistors enables the collapse of basic logic functions into compact implementations with reduced area relative to CMOS counterparts. Thereby, a key aspect is the exploitation of the efficient XOR and wired‐AND functionality inherent to multi‐gated reconfigurable devices [121]. This property is particularly useful in arithmetic circuits, as XOR and Majority (MAJ) implement the sum and carry function of a full adder [102], as shown in Figure 7e. The circuit was first demonstrated in hardware several years later [103]. In Ref. [122] a TCAD‐based predictive model Ge NW‐based TIG‐RFETs compatible with the critical dimensions of the 14 nm FinFET technology was used to show that such a 1‐bit full adder can achieve over three orders of magnitude reduced static power, and an order of magnitude reduced dynamic power relative to CMOS counterparts, at the cost of a notable increase in delay. Another design that avoids carry propagation in a pass‐transistor‐logic configuration was proposed later [123], thereby substantially reducing propagation delay. When used to implement a 32‐bit Ripple Carry Adder (RCA), this design results in 4% the worst case energy‐delay‐product (EDP, J×ps) compared to a 14 nm FinFET implementation, whereas the design in Ref. [122] has an EDP of over five times the standard CMOS design. As compared to these simulation‐based studies, hardware implementations still lag behind. While several 1‐bit full adders have been demonstrated, only recently the first 2‐bit full adder realization has been shown in Ref. [124]. The latter, illustrated in Figure 8a, represents the largest RFET‐based circuit realized to date, with 28 TIG‐RFETs fabricated using a modified 22 nm FDSOI industrial process.

**FIGURE 8:**
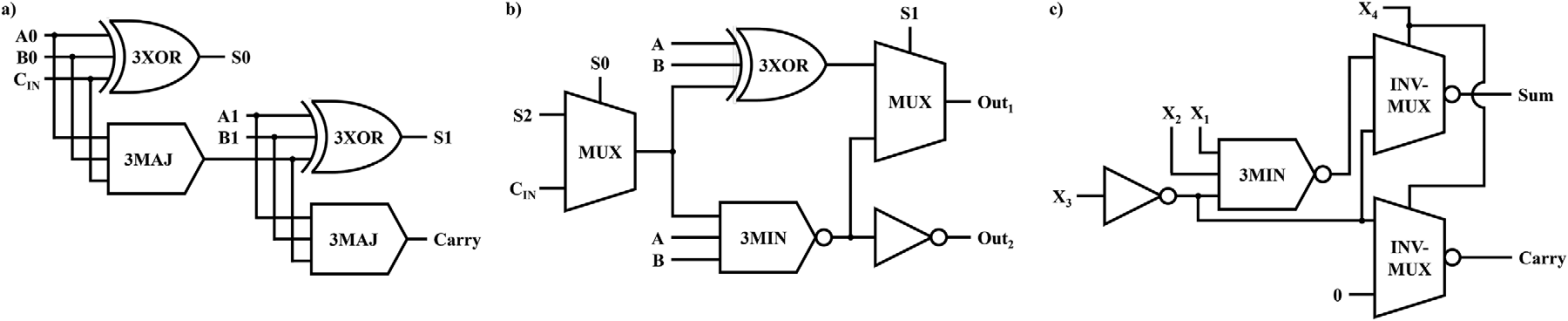
Block diagrams of efficient RFET circuit implementations using different concepts. (a) 2‐bit full adder utilizing higher‐order 3XOR and 3MAJ gates [124]. (b) Reconfigurable ALU utilizing gate‐level reconfiguration [104]. (c) Approximate 4:2 compressor design with reduced gate complexity [139]. The corresponding truth tables are provided in Figure S1.

The second design approach exploits internal functional reconfiguration. In contrast to sparse‐lookup schemes commonly used in AI accelerators, where multiple precomputed partial results are stored and selected at runtime, reconfigurable gates dynamically adapt their functionality to implement multiple computation paths within a single circuit stage. This allows the circuit to resolve its functional behavior during signal propagation, such that the effective operation is established when the input reaches the gate's critical path. As a result, the number of computational steps can be reduced, thereby lowering overall switching activity in the system. The design approach was first exemplified on a 16‐bit conditional carry adder [105]. Also, the first dense arithmetic logic units (ALUs) have been conceived [104, 125], with one implementation shown in Figure 8b. Combined with the low leakage of Schottky barrier technology, this substantially reduces overall power consumption, yielding improvements in several metrics, such as power‐delay product (PDP), even accounting for the increased delay relative to conventional inversion‐channel MOSFETs. However, to be efficiently exploited, the classical CMOS circuit must be mapped to an implementation that can be efficiently executed with reconfigurable gates. Indeed, previous work has shown that, at the synthesis level, an XOR‐Majority‐Graph (XMG) circuit‐mapping implementation better exploits these properties than standard AND‐Inverter‐Graphs (AIG), achieving up to a 12.36% area reduction independent of the technology node [97].

Beyond combinatorial circuits, some sequential circuit elements employing RFETs have been proposed [126, 127]. In Ref. [128], the authors utilized the wired‐AND functionality to introduce a True Single‐Phase Clock (TSPC) Flip‐Flop with improvement in area (20%), delay (30%), and leakage power (7%) relative to a 22 nm FinFET implementation. This design can be modified to implement a double‐edge‐triggered TSPC [129] for increased throughput. In the same work, the authors presented an SR latch capable of producing two random bits in a single clock cycle by switching between NAND‐ and NOR‐based implementations at runtime via device‐level reconfiguration (see Figure 7a,b). Another work proposed using the versatility of RFETs in combination with CMOS elements to implement a pseudo‐static random access memory (SRAM) cell capable of switching between dynamic RAM and traditional SRAM [130]. In terms of realization, the authors in [81] demonstrated a reconfigurable circuit block comprising six ${\mathrm{WSe}}_{2}$‐based DIG‐RFETs that can act as a D‐latch. Through reconfiguration, the same block can be employed for arithmetic operations such as a MUX or a 1‐bit adder.

At the system level, the intrinsic fine‐grain programmability of RFET‐based logic cells has the potential to enable efficient implementation of field‐programmable gate arrays (FPGAs) [131]. Previous reports have shown that RFETs can be used for fast and area‐efficient two‐input look‐up‐tables (LUTs) [132]. Moreover, by replacing SRAM‐controlled MUXs and LUTs with RFET‐based counterparts, it is possible to reduce the overall delay by up to 32% and the energy‐delay product by 13% compared to CMOS‐based implementations [133]. Others have proposed specific topologies to form efficient clusters [134, 135] capable of supplanting traditional LUTs. While LUT‐based architectures are typically favored in CMOS due to their speed advantages, they can also be replaced with universal‐logic‐modules (ULMs) based on the intrinsic properties of RFETs in energy‐constrained applications. However, due to the difference in expressiveness, ULM adoption for FPGAs also requires the re‐design of the overall architecture as well as an adjustment of the related EDA tools [136]. Other works have proposed RFET‐based logic elements sufficiently capable of replacing LUTs altogether, reducing the transistor count, and cutting the static and dynamic power in half [125].

### 3.3 Non‐Traditional Computing Paradigms

Beyond architectural implementations of classical digital designs, the unique properties of RFETs have also motivated their exploration in alternative computation paradigms such as approximate computing, stochastic computing, and asynchronous computing.

The approximate computation paradigm exploits certain error‐tolerant applications to trade off result accuracy for performance or energy improvements [137]. One possible implementation is through approximate compressors, a key component of multiplier circuits [138]. For example, in Ref. [139] RFETs were employed to realize a 4:2 approximate compressor, displayed in Figure 8c. It was shown that an 8×8 Dadda approximate multiplier utilizing this compressor can achieve an area and PDP reduction of 46% and 42%, respectively, when using a predictive 14 nm Ge‐RFET model as compared to a 14 nm FinFET CMOS‐based implementation.

Among the different approaches to approximate computing, stochastic representations are particularly attractive due to their ability to map complex arithmetic operations onto simple logic primitives. In this paradigm, numerical values are encoded as probabilities within bitstreams, enabling highly compact and energy‐efficient implementations. In Ref. [140], the authors show that RFETs can efficiently implement Stochastic Number Generators (SNGs) and Additive Parallel Counters (APCs), key components of stochastic computing. For example, a traditional APC RFET implementation results in gains in terms of area (18.4%), delay (5.7%), and energy (37.7%) relative to a CMOS counterpart. When used to implement a stochastic computing neural network (SCNN) accelerator, it achieves 98.5% accuracy on the MNIST dataset while consuming less energy than CMOS‐based solutions.

Reconfigurable FETs can also find applications in computation schemes beyond the traditional current or voltage‐based approaches. Among them, time‐domain computing and signal processing could be a promising alternative. In this case, the information is encoded in a timing parameter, such as a pulse width or delay relative to a reference clock, which can encode multiple bits of information, similar to traditional analog computing schemes [141, 142]. However, as the functionality is performed essentially by digital blocks, it is possible to achieve smaller areas, better voltage scaling, and utilize developed digital electronic design automation (EDA) tools [143, 144]. However, these circuits can suffer from low performance due to the relatively large times required in digital‐to‐time and analog‐to‐time conversion steps to achieve sufficient resolution [145]. Moreover, they require a stable reference clock, which is challenging at high frequencies [146]. However, the intrinsically lower drive currents of RFETs can be beneficial in this regard [147, 148], particularly in edge applications where energy efficiency and compactness are often more valuable than raw performance. The large analog tunability of RFET‐based circuits (see Sections 2 and 5) can be leveraged to efficiently implement delay‐based circuits. For example, in Ref. [149], the capability to control the drive current through the polarity gate voltage ($V_{P}$) is used to tune the delay of an RFET inverter chain. Paired with the intrinsic XNOR functionality of a single TIG‐RFET, a compact voltage‐controlled pulse‐width modulator (PWM) circuit can be realized, shown in Figure 9a. The resulting pulse width is a function of $V_{P}$ (Figure 9b), which can be used as a transducer in analog‐to‐time converters or as a building block for analog front ends.

**FIGURE 9:**
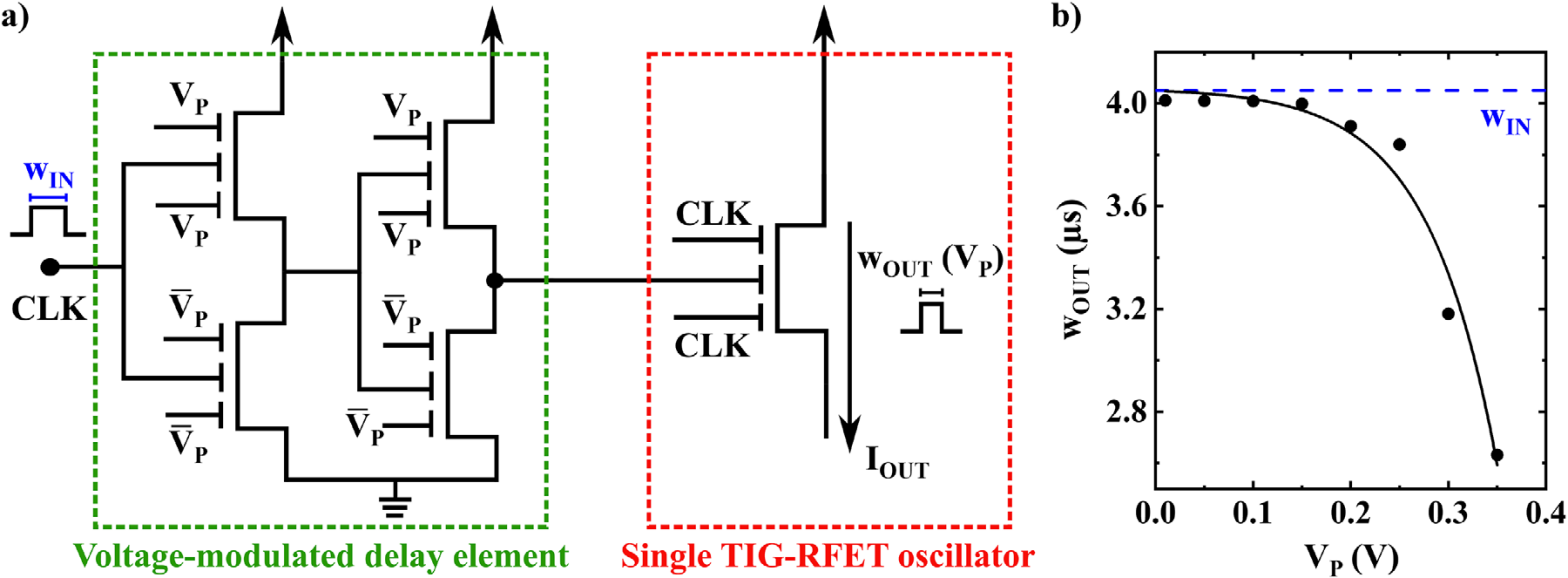
RFETs for Time‐Domain Mixed Signal Computation. (a) Schematic of a voltage‐controlled pulse width modulator (PWM) circuit, as proposed in [149]. The program voltage $V_{P}$ modulates the current driven by the coupled inverters, thus controlling the delay. CLK is an external clock signal. (b) Width of the output pulse $w_{\mathrm{OUT}}$ as a function of the program voltage. By increasing $V_{P}$, the output width is reduced with respect to the input pulse width ($w_{\mathrm{IN}}$), shown with the horizontal blue dashed line.

At the opposite end of the timing spectrum, asynchronous computing avoids the use of a global clock altogether. Most modern synchronous digital designs show growing energy demands, particularly driven by the clock signal, especially in high‐frequency applications [150]. In very scaled, GHz frequency circuits, clock management is a growing issue [151]. In this context, asynchronous designs appear as a promising alternative. Among them, null convention logic (NCL) is an approach that utilizes multi‐rail encoding to eliminate timing references [152]. There are 27 fundamental gates in NCL, which are capable of implementing all functionalities [153]. Many of them, when implemented with regular CMOS, utilize Majority, Minority, and XNOR as building blocks. The compact versions of these gates, enabled by the multi‐gated RFET technology, could enable efficient implementations. Moreover, NCL gates typically require a RESET signal, which could be provided intrinsically by the reconfiguration knob. Furthermore, some authors have proposed merging multi‐threshold logic (MTL) with NCL: multi‐threshold NCL (MTNCL) [154]. These two approaches have shown great synergy as NCL circumvents the drawbacks typically associated with MTL. Therefore, MTNCL can achieve ultra‐low‐power circuits with a smaller footprint than even traditional NCL circuits [154]. In this sense, reconfigurable transistors with tunable threshold voltages could enable extremely low‐power implementations. While TIG‐RFETs intrinsically show dual threshold depending on the operation modes, analog tuning of the threshold voltage is also possible through the back‐gate in planar implementations.

Taken together, these non‐traditional computing paradigms exploit different properties of RFETs. Approximate and stochastic computing can benefit from their compact implementation of complex logic functions, while time‐domain computing can leverage their analog tunability to control temporal quantities such as delay and pulse width. Asynchronous approaches, in turn, can exploit the intrinsic multifunctionality and tunable threshold voltage of multigate RFETs to realize compact clock‐independent logic. Despite their different operating principles, these paradigms share an emphasis on reducing energy consumption and hardware complexity rather than maximizing raw computational performance, making them particularly relevant for energy‐constrained applications. More generally, such constraints are characteristic of edge computing applications, where reconfigurable SB‐based transistors have shown significant potential [155], achieving up to 4 times lower energy dissipation on a production RISC‐V core relative to CMOS counterparts.

### 3.4 Hardware Security

Hardware security is a particularly attractive application domain for RFET‐based digital circuits because the same device properties that enable compact polymorphic logic also map directly to security primitives. In particular, runtime polarity control, multiple independently controlled gates, structural symmetry between operating modes, and compact realization of multiple Boolean functions enable security features to be embedded at the device and standard‐cell levels. This differs from conventional CMOS‐based approaches, in which logic locking, camouflaging, and side‐channel countermeasures are commonly introduced through additional gates, duplicated structures, or dedicated masking circuitry [156].

Early work on RFETs based on silicon nanowires demonstrated that emerging reconfigurable devices can support hardware‐security primitives, such as camouflaged logic and compact polymorphic gates, thereby establishing the foundation for RFET‐based secure circuit design [157, 158, 159]. Such a protection of integrated‐circuit intellectual property (IP) is one of the most direct security applications of RFET polymorphism. In conventional CMOS circuits, reverse engineering of the transistor topology, random number generators (RNGs) and physically fixed layouts can often reveal substantial information about the implemented Boolean function. In contrast, an RFET‐based polymorphic cell can retain the same physical structure while its logical function is selected through hidden polarity‐control or configuration signals [160]. Therefore, a physically observed cell does not necessarily reveal whether it is operating as a NAND, NOR, XOR, XNOR, or another supported function. This feature is particularly appealing in the field of logic locking countermeasures. Hybrid CMOS/SiNW‐FET logic‐locking architectures have been described, in which polarity‐controllable devices can be used to implement key‐dependent polymorphic logic with lower structural overhead than conventional insertion of additional CMOS XOR key gates alone [159]. Stronger obfuscation architectures based on emerging reconfigurable FETs have subsequently been investigated against reverse‐engineering and logic‐recovery attacks [161]. Logic locking has also been proposed as case‐study for a new implementation of a universal polymorphic gate topology based on RFETs, showcasing energy efficiency and high performance in securing ISCAS‐85 c6288 and c499 benchmark circuits [116].

RFET polymorphism, however, should not be considered sufficient by itself to guarantee resistance against all logic‐locking attacks. Even when the local gate function is hidden, the surrounding locked architecture must still be designed to resist oracle‐based attacks, SAT‐based attacks [162, 163], removal attacks, sensitization attacks, and machine‐learning‐assisted structural recovery. The last class of attacks has become more prominent where graph neural networks (GNNs) are used to identify the underlying structure [164, 165, 166].

Conventional fault‐analysis‐based logic encryption provides an important reference point in this context because it selects locking locations according to their ability to propagate incorrect‐key effects to observable outputs [167]. RFET‐based IP protection is therefore most compelling when the intrinsic ambiguity of polymorphic devices is combined with security‐aware locking architectures and attack‐driven evaluation.

A physically camouflaged circuit must conceal not only its Boolean function but also the physical signatures associated with different hidden configurations. For example, two RFET configurations that appear identical in layout may still be distinguishable if their propagation delays differ sufficiently under carefully selected inputs. This challenge was addressed through RFET‐based delay‐invariant logic design, where alternative reconfigurable gate configurations were analyzed under process variations. The resulting designs were shown to produce overlapping delay characteristics for different operational modes, thereby increasing the difficulty of timing‐based deobfuscation [168]. Formal analysis of camouflaged reconfigurable circuits further extended this direction by evaluating delay invariance in RFET‐based gates, combinational circuits, and sequential structures using automated analysis methods [108]. Consequently, RFET‐based IP protection should be assessed not only through functional ambiguity, but also through its resistance to timing‐based physical inference.

Reconfigurable transistors are also promising for reducing side‐channel leakage in cryptographic hardware. Earlier work on three‐independent‐gate field‐effect transistors investigated logic structures for differential power analysis mitigation, exploiting polarity control and compact circuit realization to reduce data‐dependent variations in the observable power behavior relative to conventional implementations [169]. A more direct RFET‐based countermeasure is provided in [170], which introduces reconfigurable blocks with multiple *isofunctional* configurations. In this approach, the circuit preserves the required Boolean operation while changing its physical implementation during operation, thereby reducing the correlation between secret‐dependent intermediate values and measured power traces. The method was evaluated using correlation power analysis on PRESENT and Piccolo S‐box implementations, demonstrating that RFET polymorphism can support runtime side‐channel hiding in addition to static circuit camouflaging [170].

In another approach, the authors proposed a D‐flip‐flop design utilizing TIG‐RFETs [126]. They leveraged the symmetric I–V characteristics of the devices to equalize pull‐up and pull‐down currents. This modified true single‐phase clock design minimizes supply current variation to under 1%, successfully masking information leakage against correlation power analysis. Lastly, the reconfigurability features of RFETs have been used in True‐PolyTronik [171] to enable automated fault‐injection detection and subsequent reconfiguration of a circuit under attack to an invalid functionality. Based on these works, RFET‐based designs for dedicated cryptography primitives, such as random number generators (RNGs) and physical unclonable functions (PUFs), have emerged. PUFs represent another important security primitive for which reconfigurable transistor concepts are attractive. A configurable ring‐oscillator PUF based on a reconfigurable FET has been proposed to exploit both process‐induced device variability and electrically selectable transistor configurations [172]. In comparison with a fixed ring‐oscillator PUF, such reconfigurability can enlarge the available challenge‐response space without requiring a proportional replication of oscillator structures. This property is particularly attractive for lightweight authentication in area‐ and energy‐constrained embedded systems. However, RFET‐based PUFs require careful evaluation of uniqueness, reliability, uniformity, voltage and temperature sensitivity, aging stability, and resistance against machine‐learning‐based modeling attacks before they can be considered mature hardware roots of trust.

TRNGs are essential components of hardware roots of trust because they provide entropy for cryptographic keys, nonces, initialization vectors, and challenge generation. In principle, RFET devices offer several properties that could be useful to provide compact entropy‐source designs, including polarity‐dependent conduction, configurable delay paths, bias‐sensitive switching characteristics, and reconfigurable oscillator structures. These properties could potentially be exploited in TRNG architectures based on oscillator jitter, metastability, or dynamically selected noisy paths. A case study already showcased that a metastable dynamic gate reconfiguration can be exploited to design a PUF with high throughput [129, 173, 174]. Encryption exploiting RFET‐based architectures has also been explored to yield secure logic circuits [175] as well as multi‐state communication protocols [176].

The relevance of RFET‐based security becomes stronger when polymorphic functionality is considered together with industrial technology compatibility and low‐voltage operation. Recent RFET devices processed on industrial FDSOI technology demonstrates that security‐oriented polymorphic circuits can be realized using a platform compatible with an advanced integrated‐circuit fabrication [177]. Furthermore, ultra‐low‐voltage RFETs implemented in 22nm FDSOI technology have been demonstrated at a supply voltage of 0.8V, together with a dynamic circuit‐obfuscation concept based on a merged adder/subtractor structure [30]. Such results are particularly relevant for embedded and edge‐security systems, where conventional CMOS protection mechanisms may impose significant area, timing, or power overheads.

## 4 Post‐Shannon Computation

### 4.1 RFETs for Memory

Post‐Shannon computing summarized all types of computation that go beyond the classical limits described by Claude Shannon's theory of information and communication. With the emergence of artificial intelligence, an increasing number of studies have begun to explore how RFETs can be utilized in such circuit designs that go beyond digital, including computing‐in‐memory (CIM), analog‐in‐hardware computation, and neuromorphic computing. Typically, in such designs, computing and memory resources are integrated, mitigating the infamous von‐Neumann bottleneck.

A first use case for RFETs in memory design is their use as selector devices for DRAM, which require ultra‐low off‐state leakage to maximize data retention and minimize refresh cycles. Due to the multigate structure, RFETs can achieve these ultra‐low‐leakage requirements during standby mode while instantly reconfiguring into a highly conductive state for read and write operations. Beyond the traditional 1T1C architecture, especially a capacitorless 2T DRAM structure has been explored in simulation studies, allowing for a potential low‐cost, high‐density embedded memory alternative [178]. To circumvent tunneling‐induced recombination that degrades the memory state, the author proposes intentionally misaligning the drain‐side gate to shift the charge‐storage region away from the drain‐side junction. A three‐gated planar RFET structure where the potential well for hole storage can be inherently isolated from the Schottky junctions by the ungated regions was also simulated [179]. This proposed multi‐gated RFETs demonstrates superior retention compared to the earlier discussed two‐gated counterpart. Furthermore, the dimensional scaling constraints of three‐gated RFETs have also been evaluated [180]. In Ref. [181], the authors transition this RFET 1T‐DRAM into the 3D domain by evaluating a nanowire gate‐all‐around (NW GAA) RFET, where the operational voltage levels were optimized to reduce energy consumption to the fJ range with high immunity to BL disturbances, required for ensuring data integrity in dense embedded memory arrays.

RFETs have also been discussed as an alternative for next‐generation hardware caches owing to the physical scaling limitations and leakage constraints facing sub‐micron CMOS technology [182]. Providing both *n*‐ and *p*‐type functionality, RFETs enable the design of 6T‐SRAM cell from a single device, albeit needing a accumulation of oxide traps over time. This shift from CMOS to RFET‐based SRAM is explored by Ref. [183], where the baseline stability of a planar RFET‐based SRAM is established. Reference [184] examines the impact of radiation on the stability of these cells by measuring the shift on the threshold voltage for both *p* and *n*‐type polarities. It is observed that symmetrical RFETs exhibit an imbalance between read and write stability, which radiation exacerbates. Recognizing this imbalance, in Ref. [185] the incorporation of an asymmetric drain‐side spacer was suggested that enhances the driving strength of the pull‐down transistor during read operations without penalizing write operations. Furthermore, departing from the planar optimizations, Ref.[186] explored the 3D domain by simulating TIG‐RFETs built with vertically stacked silicon nanowires, which predicted significant reductions in leakage power and average power. However, the drawbacks of the RFET‐based design with regard to the cell footprint, voltage swing, and speed outlined in Ref. [183] prevent it from currently serving as a true alternative to CMOS‐based SRAM.

### 4.2 Embedded Non‐Volatility

Most of the designs discussed so far utilize a volatile voltage signal to define the polarity of the RFETs. However, to retain the device program after a power down, store a security key for hardware security applications, or successfully leverage their functions for in‐memory computing (IMC) and neuromorphic designs, RFETs have to be combined with a non‐volatile storage element. This need has led to a number of works aimed at achieving a direct monolithic integration of the RFET core with a nonvolatile memory element. Several non‐volatile RFET cell concepts have been demonstrated in the literature, based on mechanisms such as charge trapping, floating‐gate storage, and ferroelectric polarization.

In recent years, RFETs featuring an integrated ferroelectric layer have garnered particular interest due to their ability to enable ultra‐low power IMC architectures achieving sub‐nanojoule energy consumption per programming event, as elucidated in Ref. [187]. Other works in Ref. [46] further explain that by integrating an ${\mathrm{Hf}}_{x}{\mathrm{Zr}}_{1-x}O_{2}$ layer with dual top‐gated silicon RFETs, the polarization charges can be employed to modulate the height of the two Schottky barriers, directly influencing the carrier injection behavior. This allows the selection of a multitude of well‐separated current levels by applying intermediate voltage pulses.

In another approach, Ref. [8] used single‐walled carbon nanotubes (SWCNTs) integrated with aluminum scandium nitride (AlScN) as the ferroelectric gate dielectric. These devices demonstrate well‐balanced on‐state currents of approximately 270 $\mu A\;\mu m^{-1}$, on/off ratios exceeding $10^{5}$, with large memory windows of 0.26 $V\;{\mathrm{nm}}^{-1}$ and 0.08 $V\;{\mathrm{nm}}^{-1}$ in the hole and electron conduction regions, respectively, and retention upto 2 days. Authors in Ref. [6] demonstrated quasi‐nonvolatility via reconfigurable silicon transistors that feature a p–n–p–n structure with a single‐gated region. The device uses a positive feedback mechanism to physically trap charge carriers in the potential wells formed by the p‐ and n‐regions. These trapped carriers are retained during standby, thus preserving the logic state for an extended period. Both studies discuss the potential of these devices for ternary content‐addressable memory (TCAM) cells, presenting a massive reduction in footprint compared to conventional CMOS.

Advancements in 2D materials further expand the capabilities of Fe‐RFETs, as demonstrated by Ref. [188], who used a 2D ferroelectric material, ${\mathrm{CuInP}}_{2}S_{6}$ (CIPS), in conjunction with the channel material ${\mathrm{MoTe}}_{2}$. In these heterostructures, nonvolatility arises from the alignment of dipoles within CIPS under an electric field. Additionally, these devices exhibit a tunable photoresponse dependent on their programmed nonvolatile state. This concept has been applied using an asymmetric ferroelectric‐gated ${\mathrm{WSe}}_{2}$/CIPS homojunction for in‐sensor artificial visual systems with a successful demonstration, recording an ultrahigh rectification ratio of 2 × $10^{6}$, a near‐ideal diode factor of *n* = 1.45, and reconfigurable bidirectional photoresponse controlled via a single gate [189]. The authors in Ref. [39] proposed an alternative 2D RFET structure where an ${\mathrm{Al}}_{2}O_{3}$‐encapsulated black phosphorous (BP) layer was used as the channel material to address the challenge of weak polarization performance associated with CIPS. The non‐volatility of these reconfigurable devices was successfully demonstrated as the device retained the band‐bending and consequently, its polarization state on removal of the applied positive and negative programming voltages. This device has been further employed for fuzzy logic‐enabled edge computing, where it enhanced the recognition accuracy of pneumonia CT scans by 10% compared to standard CNNs [39].

Alternative methodologies for memory integration within the logic unit propose using charge‐trapping similar to conventional Flash technology. Authors in Ref. [45] discuss the integration of a charge trapping metal‐nitride‐oxide‐silicon stack into a Si nanowire transistor. The devices yield an on/off ratio of $10^{6}$ and a memory window of 0.25 and 0.35 V for *p*‐type and *n*‐type nonvolatile operation, respectively. In Ref. [190] the authors demonstrated a ${\mathrm{WSe}}_{2}$‐based partial floating‐gate FET. The potential at the Schottky barriers is set by the partial floating SG and DG located beneath the channel, while the device is controlled by a FG and an additional source‐side control gate. This allows the device to operate as either a reconfigurable transistor or a reconfigurable non‐volatile memory, which is exploited to realize linear and non‐linear logic gates for in‐memory computing. Other demonstrations of memory integration via a floating gate are depicted in Ref. [191], where a graphene‐based memory platform using DIG‐RFETs was presented. These devices exhibit robust operation over a temperature range of 10 to 300 K, endurance beyond 9800 cycles, and a memory window of 72 V under a 40 V back‐gate voltage. Other works presented an inverted scheme, where the floating gate is positioned on the opposite side of the channel, circumventing the parasitic series capacitor that often reduces gate‐to‐channel coupling [192]. These devices also demonstrated a wide memory window of 60 V and a spiking energy of 150 pJ. Interestingly, owing to its approximately 10‐min retention time, this was proposed as a security feature that allows sensitive data stored in it to be erased after a set duration. However, while these concept demonstrations are valuable, lowering the operating voltage while increasing endurance and retention will be crucial tasks for non‐volatile RFET designs making their way into applications such as those explained in the next section.

### 4.3 Analog In‐Hardware Computation

The proliferation of modern data‐centric applications relies heavily on highly parallelized matrix‐vector multiplications (MVMs) [193, 194]. However, executing these MVMs on traditional von‐Neumann architectures incurs severe energy consumption and latency bottlenecks due to data shuffling between physically separated computational and memory units. To overcome this bottleneck, there is a critical need for advanced nanoscale devices capable of highly parallel, low‐power operations directly in hardware, often employing new ways of computation, e.g., utilizing the analog current domain or time domain instead of binary voltage levels [195, 196].

Schottky barrier‐based FETs are well‐suited for in‐hardware computation approaches, particularly at the edge, where energy consumption is a significant constraint. The high on‐off current ratio achieved with RFETs, at overall lower currents typical of SB‐FET devices, results in ultra‐low power dissipation [197]. At the same time, the high expressiveness of the devices can be used to efficiently map CIM base‐functions. For example, binary neural networks (BNNs) use the XNOR function and bit counting to perform operations [198], significantly reducing hardware overhead while preserving accuracy. Multi‐gated RFETs, with their intrinsic XNOR functionality, can be used to implement efficient BNN [199], especially when combined with a non‐volatile storage element.

The addition of a third logic state results in ternary neural networks (TNNs), which have shown increased accuracy [200, 201] and reduced number of operations [202]. Reconfigurable FETs have been shown to be capable of this multi‐valued logic. In Ref. [203], the authors utilized a MoS_2_‐ based device with front and back gate contacts, which, depending on the steering mode, yields different threshold voltages and subthreshold swings, to realize a ternary cell with only two transistors. Moreover, in Ref. [204], the authors demonstrated multivalued logic using a single ${\mathrm{MoTe}}_{2}$ transistor with a top central gate and a back gate. In this case, depending on the biasing combinations of the two gates, it is possible to enable either high, intermediate, or low current states, realizing three distinct logic levels. In both cases, the cells can be reconfigured between binary and ternary logic functionalities at runtime, allowing optimization in different applications. Another method to realize ternary logic cells is to employ devices that exhibit negative differential transconductance (NDT) [9]. While this property can be realized with complex materials or device structures, it is also enabled by the anti‐ambipolar mode of multi‐gated RFETs [205].

The capabilities of RFETs are fully leveraged in analog applications beyond three‐level logic. Due to their high tunability, several applications for in‐hardware computation have been identified. Gated Schottky Diodes and RFETs have been shown to possess large linearity in the channel conductance as a function of the front gate voltage [206, 207], particularly for hole conduction. This allows for linear mapping of values for in‐hardware computation, avoiding complex conversion operations [208]. Further, previous work has shown an almost linear relationship between the current and the source‐drain potential when the voltage is applied at the injecting terminal [209]. This bi‐linearity can be used to efficiently implement multiply‐and‐accumulate (MAC) operations, which typically account for the bulk of operations and energy consumption in Neural Network implementations [210]. In Ref. [48], the authors employed the bi‐linearity of back‐biased planar RFETs to implement the MAC operation directly in hardware, obtaining the same accuracy as the in‐software solution, but with an order of magnitude less power dissipation. Interestingly, the additional tuning enabled by the FDSOI technology through the back gate allowed the implementation of addition directly at the device level, which has the potential to further simplify analog implementations by avoiding current adders or conversion steps between the digital and analog domains [48, 211].

The bi‐linearity of multi‐gated RFETs has so far remained unexplored. In this case, the added flexibility could be leveraged for adaptable circuits, capable of operating both as XNOR functions for quantized NN and in a fully analog manner for multi‐bit precision. A similar approach has been implemented in a reconfigurable transistor with a ${\mathrm{WSe}}_{2}$ channel [212]. In this case, the back gate is utilized to linearly tune the conductance (i.e., the weights) while the inputs are applied at the front gate. When the values are continuously modulated, a high‐precision artificial neural network (HANN) can be realized, achieving accuracy comparable to that of a silicon implementation in obstacle detection tasks. Conversely, when the input and weights are limited to $\pm V_{\mathrm{DD}}$, the resulting binary network shows increased energy efficiency at the cost of a 3% decrease in accuracy. The tunability of SB‐based devices has also been proposed as a building block for fuzzy layers in the hardware implementation of fuzzy logic, achieving a 10% accuracy improvement relative to a standard CNN in CT (computed tomography) image recognition tasks [39].

Reconfigurable transistors with an integrated non‐volatile element are particularly promising for content‐addressable memories (CAMs). Unlike standard memories that require an address to retrieve data, CAMs accept a data input (a query) and simultaneously compare it against all stored entries in a single operation [213]. This inherent parallelism is ideal for scenarios where rapid, one‐shot matching is required. A schematic example is shown in Figure 10, utilizing a back‐bias RFET with a ferroelectric layer at the back gate. Depending on the orientation of the ferroelectric dipoles in the layer, the ambipolar transfer characteristics will be shifted [27, 209, 214], as described in Section 2. A positive polarization (shown in Figure 10a results in an n‐type curve within the working range, defined as the stored “1” state. Conversely, negative polarization yields a p‐type channel, which is defined as the “0” state. The input search voltage is applied at the top gate ($V_{G}$), appropriately selected close to $V_{\mathrm{DD}}$ and GND to search for “1” and “0”, respectively. As shown in Figure 10b, the device will only conduct a significant current when there is a match between the stored and search states. This device can be integrated in a crossbar array to implement a binary CAM with only a single device per bit (Figure 10c). This approach has been implemented using a ${\mathrm{MoTe}}_{2}$ channel and a CIPS charge‐storage layer [120]. By combining multiple transistors, it is possible to perform multi‐bit matching in a single operation and compute the hamming distance (HD) between the query and the stored state.

**FIGURE 10:**
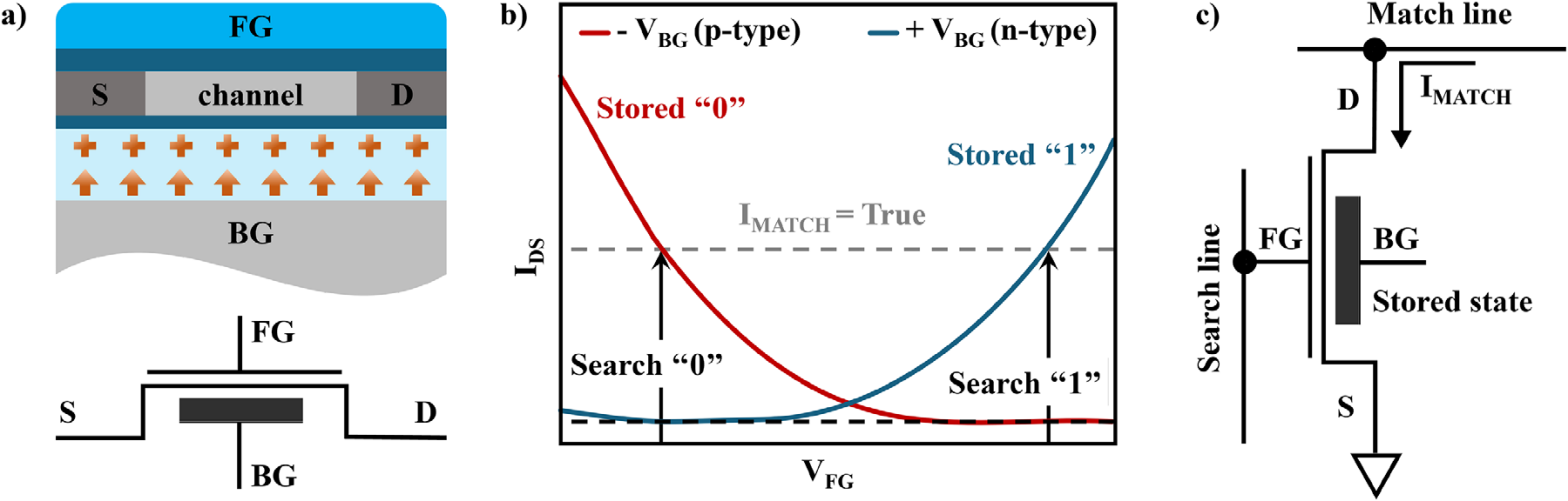
RFETs for Ternary content‐addressable memory (TCAM). (a) Schematic of a BB‐RFET structure incorporating a ferroelectric oxide layer controlled by BG, whose polarization determines the electrostatic doping state of the channel. (b) Schematic linear transfer characteristics. Positive ferroelectric polarization shifts the ambipolar transfer characteristics toward *n*‐type behavior within the operating window, defining the stored logic state “1”. Negative polarization induces a shift toward p‐type behavior, defining the stored logic state “0”. The search operation is performed by applying a voltage to the front gate ($V_{\mathrm{FG}}$), typically biased near $V_{\mathrm{DD}}$ for a “1” search or near GND for a “0” search. A significant conduction current occurs only when the applied search voltage matches the stored polarization state, enabling intrinsic match/mismatch discrimination. (c) Schematic of the device integrated into a crossbar architecture to implement a binary CAM, requiring only one RFET per stored bit.

When using multi‐state RFETs, such as TIG‐RFETs [215] or feedback transistors [216], it is possible to realize cells that conduct for any query or never conduct (remain insulating). Such configurations can provide the third “X” wildcard state required by TCAMs, provided that their response corresponds to a match for both possible query values. Thus, an always‐conducting state acts as a wildcard when current encodes a match, whereas an always‐insulating state can fulfill this role when current encodes a mismatch. This approach has the advantage that it only requires the storage of binary values while yielding ternary states, simplifying the memory overheads of other multi‐level cells like analog CAMs. A possible implementation of the wildcard insulating state is a TIG‐RFET cell with the SG and DG biased at opposite polarities, while the search query is applied at the central gate. In this case, all queries will give a low current state. An experimental implementation of a TCAM cell based on two non‐volatile RFETs was recently reported in Ref. [217]. In these devices, charge trapping in an ${\mathrm{Al}}_{2}O_{3}$/${\mathrm{HfO}}_{2}$/${\mathrm{Al}}_{2}O_{3}$ dielectric stack non‐volatily programs a ${\mathrm{MoTe}}_{2}$ channel into either n‐ or p‐type operation. Two RFETs were connected in series or parallel to realize NAND‐ and NOR‐type associative‐search cells, for which high and low currents were defined as the respective match conditions. The measured match and mismatch currents were separated by more than two orders of magnitude, and a search time of 10μs was experimentally demonstrated for the NAND‐type cell, although the authors attributed this timescale primarily to the RC delay of the measurement setup. Equal‐polarity configurations encode the binary states, whereas opposite‐polarity configurations were identified by the authors as the “X” state. However, according to the reported truth tables, the opposite‐polarity configuration produces a mismatch for both binary queries and therefore behaves as a query‐independent reject state rather than as the conventional TCAM wildcard, which must match both queries. Nevertheless, the work experimentally demonstrates compact non‐volatile RFET‐based associative‐search cells and a third functionally distinct cell response, which could be incorporated at the architecture level.

Beyond binary exact matching, the multi‐state programmability of RFETs could also support approximate searches based on the multi‐state hamming distance (MSHD). In contrast to binary HD, MSHD compares vectors whose individual dimensions may represent more than two states or symbols and counts the number of dimensions in which the stored and queried symbols differ. A corresponding multi‐state hamming distance‐based content addressable memory (MHCAM) architecture was proposed using FeFETs [218]. Whereas implementing (s) possible states through one‐hot encoding requires (s) TCAM cells per dimension, MHCAM represents them using only $\left(\left\lceil \log_{2}s\right\rceil \right)$ two‐FeFET CAM cells. Match‐line voltage scaling then allows the same array to perform either exact matching, for which the MSHD must be zero, or threshold matching, in which entries are accepted when their MSHD does not exceed a selected threshold. The latter operation enables partial or approximate matching by tolerating a configurable number of symbol mismatches instead of rejecting every entry that is not identical to the query. This capability is particularly relevant when processing noisy or naturally varying data, as demonstrated for DNA and protein pre‐alignment filtering, for which the proposed MHCAM achieved (3.8×) and (4.7×) speedups and (1.7×) and (1.8×) energy‐efficiency improvements, respectively, compared with a two‐FeFET TCAM implementation. Although the proposed architecture was evaluated using FeFETs, RFETs with multiple non‐volatile operating configurations could provide analogous compact symbol encoding and electrically reconfigurable matching thresholds.

### 4.4 Neuromorphic Computation

Neuromorphic computing relies on massively parallel interactions between artificial synapses, which store and update connection weights, and neurons, which integrate incoming signals to generate output spikes or analog responses. Efficient neuromorphic architectures place memory and computation in close proximity to reduce the power and latency overhead of data transfer between physically separated memory and processing units in von Neumann systems [219]. In this context, Schottky Barrier FETs (SBFETs) and Reconfigurable FETs (RFETs) are promising building blocks: as low‐current device architectures with reduced parasitic resistance and mitigated short‐channel effects, they can support highly parallel spiking and rate‐coded workloads while enabling hardware primitives that mimic synaptic plasticity, event‐driven signaling, and fine‐grained parallel information processing [220, 221].

At the device primitive level, SBFETs and RFETs provide several properties directly suited to neuromorphic operation. Barrier‐height or barrier‐width modulation allows ultra‐low‐current operation over a wide conductance range; polarity reconfiguration controls the sign or operating mode of the effective channel; embedded nonvolatile elements enable local storage of synaptic weights; and independent terminals allow decoupling of learning, gating, and sensing paths from the main signal conduction channel.

These properties can be co‐integrated within compact multi‐terminal devices, as in van der Waals (vdW) material platforms, where atomically sharp interfaces and strong electrostatic control facilitate reproducible analog conductance modulation and low‐leakage weight storage. Ferroelectric SBFETs exemplify this compact integration of neuromorphic functionality. Modulation of source/drain injection barriers via ferroelectric polarization switching has already been proven for Si nanowire SBFETs with ${\mathrm{HfO}}_{2}$ based ferroelectrics [222] and top‐down processed SOI‐SBFETs with ${\mathrm{Hf}}_{0.5}{\mathrm{Zr}}_{0.5}O_{2}$ ferroelectrics [223] with single gates showing non‐volatile adjustment of the on‐currrent over a range of various orders of magnitude. By employing a DIG‐RFET design with independent ferroelectric gating of the individual junctions (SG and DG), non‐volatile conductance programming with analog tunability and sub‐fJ energy per update event was shown. Both excitatory and inhibitory postsynaptic current (EPSC/IPSC)‐type responses, long‐term potentiation and depression, and spike‐timing‐dependent plasticity (STDP) have been shown in such devices, with operation energies on the order of 2 fJ per EPSC spike and stable conductance modulation over more than 10 ^5^ programming pulses [224]. A schematic of a DIG‐RFET variant following a similar approach is shown in Figure 11. In this implementation, realized in Ref. [117], the DG can act either as a modulatory neuron, as shown in Figure 11a, or as a second pre‐synaptic neuron through the wired‐AND functionality. By applying pulses at the SG, either potentiation or depression can be achieved, with negative and positive voltages, respectively, in the case of p‐type mode as illustrated in Figure 11b. Similar approaches can be realized by simmultaneously programming both SG and DG, and employing a back‐gate for device control [46, 225]. Recently, a concept involving a ferroelectrically steered floating gate on an SOI SBFET [226] demonstrated highly symmetric long‐term potentiation and depression states as a function of the number of pulses.

**FIGURE 11:**
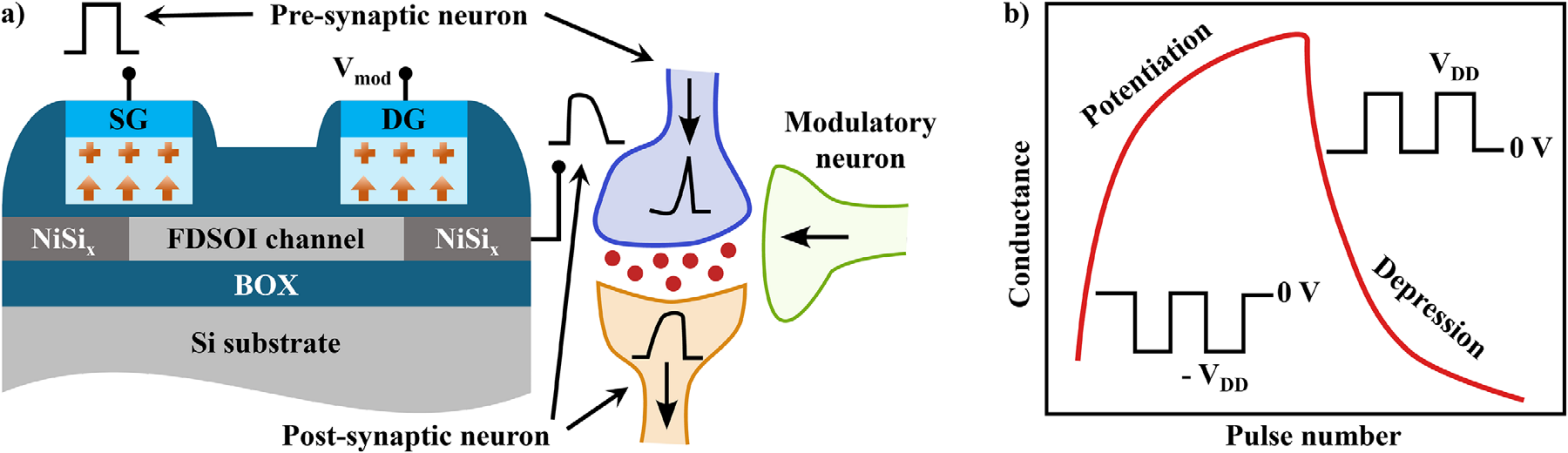
RFETs for Neuromorphic Computing. (a) Schematic of a two‐independent gate RFET‐based heterosynaptic device, following the approach in Ref. [117]. A voltage applied at the drain gate (DG), $V_{\mathrm{mod}}$, modulates the effect of the voltage applied at the source gate ($V_{\mathrm{SG}}$) on the conductance. When pulses are applied to the source gate, partial ferroelectric switching occurs, modifying the conductance through the modulation of the injecting SB. This system can effectively approximate the behavior of a biological synapse, as shown in the figure on the right. (b) Schematic of the conductance versus the number of pulses. When the device is programmed as a p‐type device ($V_{\mathrm{DS}}<0\;V$ and $V_{\mathrm{mod}}<0\;V$), negative voltage pulses increase the channel conductance (potentiation) by progressively thinning the SB for holes. Negative pulses have the opposite effect (depression). Setting the device as an *n*‐type transistor reverses the role of positive and negative pulses. Note that the same device can also be used to implement a synapse with two pre‐synaptic neurons. In this case, a second input potential pulse is applied at the DG.

Similar synaptic‐weight primitives have been demonstrated in other memory‐enabled reconfigurable transistors, including charge‐trap, floating‐gate, and hybrid polymer gate‐stack devices, which exploit trapped charge or dipolar reorientation to retain analog conductance states and emulate diverse forms of synaptic plasticity [192, 227, 228]. In van der Waals (vdW)‐based floating‐gate neuromorphic transistors, for example, 2D semiconductors coupled to engineered trapping layers provide multi‐level nonvolatile states with high retention and low‐energy programming, making them attractive artificial synapses for in‐memory neuromorphic arrays; vdW‐engineered one‐transistor–one‐ferroelectric‐memristor (1T1M) cells have demonstrated high resistance tunability, low sneak currents, and energy‐efficient neuromorphic array operation [229]. Polarity reconfiguration further extends the functional richness of a single synaptic element. Short‐channel vdW synapse devices based on ambipolar WSe2 have demonstrated electrically switchable excitatory and inhibitory behavior within a single transistor, enabling flexible mapping between hardware synapses and network connectivity patterns [230]. Related concepts have been implemented in single‐gate graphene synapses using charge trapping and ionic or capacitive gating, as well as in ambipolar oxide thin‐film‐transistors (TFT) in which gate‐bias polarity selects the dominant carrier type and thus tunes the effective synaptic response [231, 232]. Across these platforms, reconfigurability allows continuous or discrete tuning of synaptic polarity and weight over $10^{2}$–$10^{4}$ conductance levels, which is essential for implementing multi‐bit synapses, homeostatic plasticity, and on‐device learning [230, 231, 232].

The intrinsic tunability of RFETs enables compact implementations of synaptic primitives, often directly at the device level. Importantly, this neuromorphic functionality can coexist with other operating modes already discussed throughout this review. In Ref. [81], for example, a DIG‐RFET employing two localized bottom gates, SG and DG, was used to implement several digital and arithmetic functions (Section 3.2) and, when combined with a volatile capacitive node, could also realize a synaptic primitive switchable between Hebbian and anti‐Hebbian learning rules. A similar form of cross‐domain polymorphism has been demonstrated in optoelectronic devices. RFETs based on photoresponsive 2D materials can be electrostatically reconfigured between photodetection (Section 5.3) and ambipolar synaptic operation exhibiting long‐term potentiation and depression [233]. In related devices, illumination itself can modify the operating mode, for example by inducing hysteretic behavior in a device that otherwise exhibits *n*‐type transistor operation in the dark [234]. These demonstrations show that synaptic behavior need not constitute an isolated device function, but can instead form one operating mode within a broader reconfigurable hardware platform.

From this perspective, neuromorphic computation adds an additional dimension to the polymorphism of RFET‐based hardware. Device and circuit concepts discussed elsewhere in this review for logic, arithmetic, memory, and sensing can, in suitable implementations, be extended or reconfigured to provide synaptic functionality without requiring an entirely separate device technology. The significance, therefore, lies not only in realizing compact artificial synapses but also in the possibility of reusing the same physical hardware substrate across conventional computation, sensing, and adaptive neuromorphic operation. Such cross‐domain reconfigurability is particularly attractive for systems in which the required functionality changes with the operating context and provides a pathway toward highly integrated hardware that combines deterministic computation with adaptive and learning‐capable functions.

## 5 Analog and Mixed‐Signal Circuit Design and Applications

### 5.1 Tunable Analog Building Blocks

Complex analog and mixed‐signal integrated circuits (ICs) are methodically constructed of several subcircuits (i.e., functional building blocks), which are usually made up of one or more single transistors. Usually, these functional building blocks perform only one primary function, such as providing a constant voltage or current, amplifying an input signal, or copying a current. Therefore, several functional building blocks, are combined to generate a more complex circuit function, such as operational amplifiers, comparators, filters or analog to digital converters (ADCs). A fundamental challenge of designing analog and mixed‐signal ICs with nanoscale CMOS is the variation of transistor properties, such as thresholds, mobility, or output resistance, which leads to adverse circuit‐level effects, such as offsets, gain errors, and increased non‐linearity. In classical CMOS, these effects are set after the circuit layout is fixed. Thus, avoiding these practical degradations of circuit performance is often cumbersome and is typically achieved by increasing the overall circuit complexity with additional circuit elements. RFETs offer, through the inherent ability to use polarity modulation to influence transistor behavior, a significantly more powerful lever for the circuit designer than ever before. The added transistor control comes in multiple levels: on the most fundamental level, the transistor can be switched between *n*‐ or *p*‐channel, with the consequence that the polarity of the bias and supply voltages can be inverted, or e.g., the effective source/drain roles or current‐flow direction can be reconfigured using a suitable electric control. This concept was demonstrated at the operational‐amplifier (op‐amp) level, where RFET‐based Miller and open‐drain op‐amp topologies were simulated under both normal and inverted supply conditions, showing asymmetric analog blocks can be reconfigured between source‐ and sink‐type operation, and that two open‐drain RFET op‐amps with different biasing conditions can be combined to form a push‐pull‐capable amplifier [235]. Such a concept could potentially be exploited for rail‐to‐rail input operation, because the same RFET‐based input pair may be reconfigured between *n*‐ and *p*‐channel operation depending on the input common‐mode level.

Beyond polarity reconfiguration, RFETs provide a second level of tunability that arises from their multi‐gate architecture. The additional electrostatic control terminals decouple functional programming from the primary steering signal at the control gate, allowing the device electrostatic potential profile to be adjusted after fabrication. As a result, the effective conductance and transconductance of the RFET can be continuously tuned by the polarity gate or the back gate (BG) bias, leading to systematic modulation of the transfer and output characteristics. This post‐fabrication adaptability enables electrical control of key analog figures of merits (FoMs). Recently, different key analog FoM for TIG‐RFETs integrated into 22 nm FDSOI technology have been discussed [236]. The influence of both the polarity gate and back bias as tuning inputs on these FoMs has been evaluated. The analyses demonstrate that the program voltage ($V_{P}$) systematically shifts the $g_{m}/I_{d}$ characteristics toward higher $I_{D}$, indicating that comparable transconductance efficiency can be maintained at higher current levels and providing a hardware foundation for runtime‐tunable analog design. Furthermore, back‐bias can induce threshold voltage ($V_{\mathrm{th}}$) shifts while $g_{m}/I_{d}$ variations remain predictable, providing an effective solution for compensating $V_{\mathrm{th}}$ mismatches. A direct application of this tunability is found in amplifiers and current mirrors. In programmable amplifiers and current mirrors, key operational parameters, such as the amplifier gain and the current copy ratio, are no longer fixed by physical transistor dimensions but can be electrostatically tuned across several orders of magnitude [237]. Translating this concept to the 22 nm FDSOI technology, a current mirror and a common‐source (CS) amplifier are built as shown in Figure 12b, d [236]. The absolute value of output‐to‐input ratio of the CS amplifier can be adapted by changing the $V_{P}$ voltage as shown in Figure 12e. The FDSOI RFET current mirror not only allows for the dynamic modulation of the mirror ratio, but also successfully preserves excellent fundamental analog FoMs, such as a high output resistance and finite compliance voltage as shown in Figure 12c [236, 238, 239].

**FIGURE 12:**
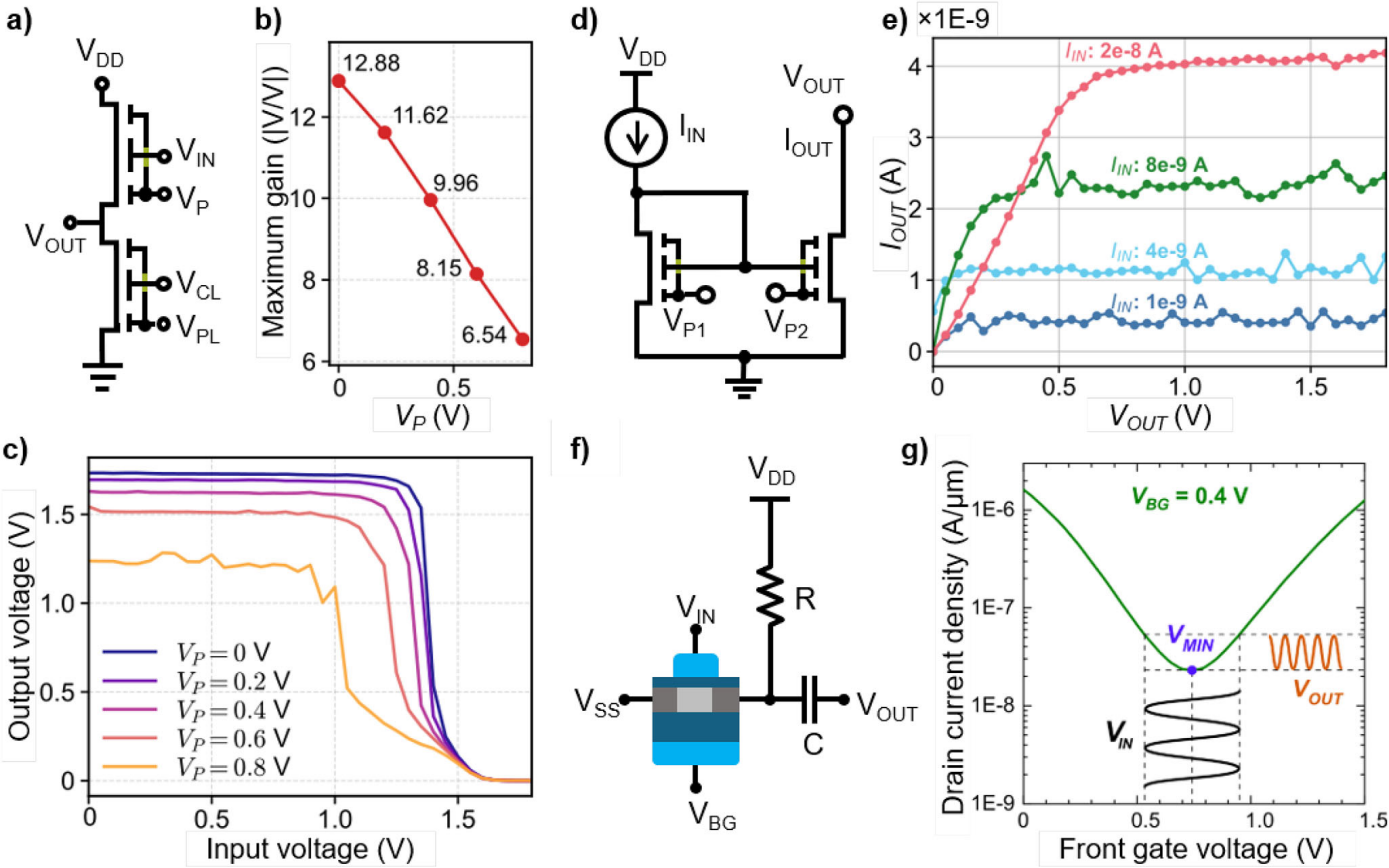
RFETs for Analog Design. A polarity‐gate‐tunable amplifier with (a) circuit diagram of the common‐source amplifier based on FDSOI TIG‐RFET, where the subscript L means the RFET serving as an active load. (e) Voltage transfer curves recorded at various $V_{P}$ levels. (b) Maximum voltage gain ($|dV_{\text{OUT}}/dV_{\text{IN}}|$) extracted as a function of the polarityvoltage $V_{P}$. (c, d) Current Mirror based on FDSOI TIG‐RFET [236]: (c) Schematic of the current mirror configuration. (d) Measured sink‐mode output characteristics obtained under different input currents ($I_{\mathrm{IN}}$). Ambipolarity of BB‐RFET for frequency doubling is shown by (f) experimental configuration [27] and (g) a related concept sketch of the operational mechanism showing a frequency‐doubled output signal ($V_{\text{OUT}}$) generated by applying a DC‐biased sinusoidal input ($V_{\mathrm{IN}}$) to the front gate [27].

The independent polarity control of RFETs can also enhance other fundamental analog topologies, such as differential stages. In an RFET‐based differential amplifier, the polarity gates introduce multiple new degrees of freedom: the overall amplifier gain and output swing can be dynamically tuned by modulating the bias current of an underlying reconfigurable current source. Separating the $V_{P}$ biases of the differential transistor pair enables continuous, precise input offset voltage calibration to compensate for process variations, thereby circumventing the need for conventional compensation circuits or discrete width‐scaling methods. Furthermore, substituting conventional active loads with RFETs enables the differential stage to be dynamically repurposed as a single‐balanced differential mixer, using the load polarity gate as a secondary, independent signal input [238]. Similar, output polarity controllable amplifiers have been demonstrated with ambipolar RFETs based on 2D materials [240].

Beyond general‐purpose amplification, the electrostatic barrier control inherent to these architectures offers significant scaling advantages for power‐constrained analog applications, such as active‐matrix organic light‐emitting diode (AMOLED) pixel drivers [241]. In related source‐barrier modulated structures, such as the source‐gated transistor (SGT), current saturation is governed by source electrostatics rather than conventional channel pinch‐off at the drain. This mechanism effectively shields the source barrier from drain‐induced variations, allowing devices with scaled channel lengths to maintain the high output impedance and exceptionally low saturation voltages required for stable current sources. Integrating this barrier‐controlled behavior, also known from multi‐modal Schottky Barrier Transistors [242], into analog driving elements ensures well‐saturated output characteristics while drastically reducing both the required layout area and static power consumption compared to conventional field‐effect transistors.

### 5.2 Analog Signal Processing

In addition to enabling tunable analog circuit blocks, the nonlinear and electrostatically programmable characteristics of RFETs can be exploited for direct analog signal processing. Useful nonlinearities may arise either in the transfer characteristics, as in ambipolar and anti‐ambipolar devices, or in the output characteristics, as in devices exhibiting negative differential resistance (NDR). Together with gate‐programmable Schottky barriers, these nonlinear input–output relations can directly reshape an applied waveform. Such device responses enable phase reversal, phase shifting, frequency multiplication, analog modulation, mixing, oscillation, and gate‐tunable rectification within a single device or a strongly simplified circuit.

Their ambipolar transfer characteristics allow the same device to operate on either the electron or hole branch, or near the minimum‐conductance point where the sign of the transconductance and the curvature of the transfer curve can be exploited for waveform transformation. This property has been used for phase reversal, phase shifting, frequency multiplication, and analog modulation [243]. For example, a with a pull‐up resistor and a pull‐down single back‐bias RFET can be dynamically reconfigured between frequency doubling, phase following, and phase reversal by shifting the operating point between its ambipolar, *p*‐type, and *n*‐type regimes [27]. These functions can further be exploited for binary frequency‐shift keying (BFSK) and binary phase‐shift keying (BPSK), with the carrier signal and the data‐dependent control signal applied to different gates [27]. More recent simulation‐based studies indicate that this multifunctionality is not restricted to the BB‐RFET architecture: three‐gate and dual‐doped RFET concepts have likewise been configured for phase following and reversal, frequency doubling, and tunable analog/RF operation through different electrical configurations of their control and polarity electrodes [244, 245]. Beyond RFETs, ambipolar and anti‐ambipolar transport in 2D‐material FETs enables in‐phase, out‐of‐phase, and double‐frequency sinusoidal outputs, which can be combined for multi‐bit analog transmission [47]. Related concepts have been demonstrated in ultrathin ${\mathrm{MoTe}}_{2}$ transistors, where electrical control of the ambipolar transfer characteristics enables amplitude‐, frequency‐, and phase‐shift keying (ASK, FSK, and PSK), while a double‐well transfer characteristic can additionally provide frequency quadrupling. Combining two programmed devices further enables multibit modulation with increased bandwidth efficiency [246]. In graphene‐based devices, V‐shaped or W‐shaped transfer characteristics further support high‐frequency signal processing, including reconfigurable frequency multiplication, mixers, phase shifters, and modulators. In particular, split‐gate graphene FETs have been proposed to switch between frequency‐doubling, tripling, and quadrupling modes through electrically controlled transfer‐curve shaping [243, 247]. At the circuit level, this waveform‐processing concept is also related to RFET active‐load modulation in differential stages, where the load polarity gate can serve as a second signal input for single‐balanced mixing [238].

The same electrostatic reconfiguration can also be exploited for rectification at the circuit level. Using BB‐RFETs implemented on a 22 nm FDSOI platform, a reconfigurable rectifier has been proposed that can be dynamically switched between half‐wave and full‐wave rectification, illustrating how polarity programmability can replace otherwise distinct rectifier topologies [214]. SB‐FETs are also attractive for signal conditioning and rectification because the Schottky barriers at the source and drain can be reshaped by electrostatic fields. Unlike traditional rectifiers, such as p–n diodes, that rely on chemical doping, SB‐FETs utilize electrostatic doping to dynamically reshape the band structure. By applying specific gate biases, the device asymmetrically modulates its contact barriers: one Schottky barrier is significantly thinned by the local electric field, enabling efficient tunneling injection and quasi‐Ohmic low‐resistance transport, while the opposite barrier remains thick to block reverse carrier transport [248]. This source‐drain barrier asymmetry creates an efficient unidirectional conduction channel for rectification. Switching the $V_{P}$ at SG and DG alters the carrier type and inverts the barrier profiles at both ends. Therefore, a single device can seamlessly change between forward rectification, reverse rectification, off‐state blocking, and full‐pass operation via electrostatic control [249]. These properties have been demonstrated in ${\mathrm{WSe}}_{2}$‐based Schottky junctions and related vdW heterostructures, where the rectification ratio, ideality factor, reverse leakage current, and reconfiguration speed are key performance metrics [250].

Another useful device‐level nonlinearity is negative differential resistance (NDR), which occurs over a finite bias range in which the current decreases with increasing terminal voltage, such that ($\partial I_{DS}/\partial V_{DS}<0$). This behavior differs from the negative transconductance found along one branch of a nonmonotonic transfer characteristic ($\partial I_{DS}/\partial V_{GS}<0$). When appropriately biased, the negative incremental conductance can compensate losses in a resonant circuit and sustain electrical oscillations, while the nonmonotonic current–voltage relation can also support frequency multiplication, mixing, and detection. These operating principles are well established in resonant‐tunneling‐diode oscillators and self‐mixing receivers, as well as in LC oscillators based on type‐III heterojunctions [251, 252, 253]. More recent examples include an NDR‐based frequency doubler implemented using a standard FDSOI transistor [254] and the dual‐gated p‐${\mathrm{MoTe}}_{2}$/n‐${\mathrm{SnS}}_{2}$ heterojunction reported in Ref. [255], in which the position of the NDR peak and the direction of rectification could be controlled electrostatically.

Programmable NDR has been integrated with electrostatic polarity control in Ge‐based RFET devices. A monolithic Al–Ge–Al nanowire heterostructures showed combined electron and hole conduction with a distinct, runtime‐programmable NDR mode in a single adaptable transistor [69]. The resulting device could therefore be configured for *n*‐type, *p*‐type, or NDR operation, with the latter proposed as a basis for compact multivalued logic and neuromorphic circuits. This concept was subsequently transferred to an ultrathin Ge channel implemented on an SOI platform, yielding a chip‐scale RFET that combined symmetric *n*‐ and *p*‐type operation with pronounced room‐temperature NDR [256]. Importantly, the additional gate terminals allow the position and magnitude of the NDR region to be controlled electrostatically [70]. This combination of polarity selection and programmable NDR could provide a compact device primitive for reconfigurable oscillators and nonlinear waveform‐processing circuits, although such analog functions have not yet been experimentally demonstrated with these RFETs.

Despite these promising results, RFET‐based analog signal processing still requires further optimization toward practical circuit implementation. Key aspects include reducing the polarity voltages, improving contact/barrier reproducibility, and translating initial small‐signal RF characterization into high‐frequency functional circuit demonstrations. The first small‐signal characterization of BB‐RFETs has shown that conventional equivalent‐circuit approaches can be applied to these devices, providing an important basis for RF circuit design and the extraction of frequency‐dependent device parameters [257]. Together with circuit‐level co‐integration with CMOS analog and RF blocks, these efforts will be important for translating RFET tunability into scalable analog systems.

### 5.3 Sensor Integration

Applications of RFETs in sensing have appeared only recently in the literature. For sensing applications, an exposed, ungated, or weakly gated region is generally advantageous, as it allows external stimuli, such as photons or chemical analytes, to interact directly with the semiconductor channel or junction regions. Many RFET architectures naturally provide such accessible regions between spatially separated gates, where the resulting perturbation of carrier transport can be detected through changes in the device current. Beyond the transduction mechanism itself, the reconfigurability of RFETs provides additional degrees of freedom for sensing, enabling different operating modes, electrical tuning of the device response to maximize sensitivity, and, in some cases, different sensing mechanisms within the same device. RFETs therefore offer a versatile platform in which sensing and computation can potentially be combined at the device level. Their capability for both *p*‐type and *n*‐type operation further facilitates their integration with complementary electronic interfaces.

One possible approach to optical sensing is illustrated in Figure 13. By applying appropriate biases to two spatially separated gates, *p*‐type and *n*‐type regions can be electrostatically induced within the same channel, forming a diode with a high rectification ratio, as shown in Figure 13a [188, 258]. When light is incident on the exposed channel region, photocarriers are generated and separated by the built‐in electric field of the electrostatically defined p–n junction, producing a photocurrent even in the absence of an applied drain–source voltage, i.e., a short‐circuit current, as illustrated in Figure 13b. The direction of this current depends on the gate‐bias configuration, corresponding to either a p–n or n–p junction, and can therefore be reversed electrically at runtime [189]. This electrically switchable positive or negative photoresponse can be exploited to represent signed weights for in‐sensor image processing, as illustrated schematically in Figure 13c. Furthermore, the magnitude of the generated photocurrent can be tuned through the applied gate biases, enabling continuous weight adjustment for neural‐network implementations [259]. Related optical‐sensing concepts have also been demonstrated using different RFET architectures and material systems. A ${\mathrm{WSe}}_{2}$–${\mathrm{Ta}}_{2}{\mathrm{NiSe}}_{5}$ heterostructure, for example, was operated using a single back gate, with different photoresponse modes selected through combinations of the back‐gate and drain–source voltages [260]. The device exhibited a responsivity exceeding 12 mA/W, which could be further modulated through the gate voltage. Electrically reconfigurable p–n and n–p diode operation has also been demonstrated in a single‐gated ${\mathrm{PdSe}}_{2}$/${\mathrm{WS}}_{2}$ heterostructure [261].

**FIGURE 13:**
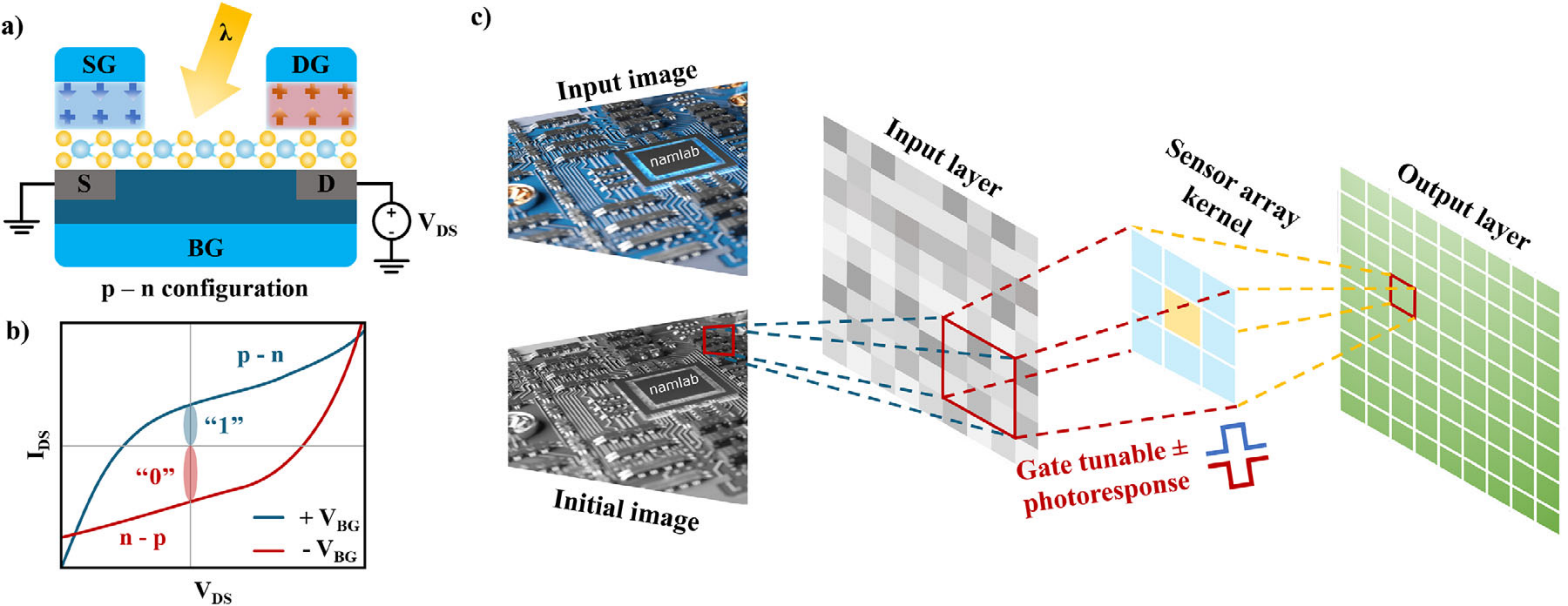
RFETs for photosensing. (a) Schematic of an RFET with two independently addressable bottom gates and a polarization layer. By independently controlling the polarization state associated with each gate, a *p*‐type channel can be induced above the drain‐side gate and an n‐type channel above the source‐side gate, forming the p–n configuration shown, or, conversely, an n–p configuration. (b) When light is incident on the exposed channel region, a photocurrent is generated even in the absence of an applied drain–source voltage. The direction of this current is determined by the built‐in electric field of the p–n/n–p junction and can therefore be reversed by changing the polarization state associated with the two gates. (c) This behavior can be exploited for neural‐network implementations, where switching between the p–n and n–p configurations determines the sign of the weights, while the back‐gate voltage, $V_{\mathrm{DS}}$, or the magnitude of the polarization can be used to tune the weight magnitude.

Beyond 2D materials, experimental silicon‐nanowire FETs have long demonstrated strong gate‐dependent optical responses, including electrically tunable photocurrent magnitude and polarity associated with Schottky‐barrier modulation [262]. Building on these properties, silicon‐nanowire RFETs have recently been proposed as CMOS‐compatible reconfigurable phototransistors, although their optical operation has so far been investigated primarily through simulation [263, 264]. The elongated morphology of one‐dimensional semiconductor nanostructures introduces an additional optical degree of freedom by giving rise to polarization‐dependent optical properties [265]. Such anisotropy has been experimentally exploited in individual silicon‐nanowire photodetectors for polarization‐sensitive detection and polarization‐resolved imaging [266, 267, 268]. This property could enable polarization‐sensitive operation in future nanowire‐based RFET photodetectors.

The versatility of RFETs can also be exploited to provide advanced functionalities in the field of chemical sensing [269]. Even though the application of silicon nanowires with Schottky barriers to chemical sensing have been demonstrated a decade ago to pH [270], proteins [271], and nanodroplets [272], building biosensors with nanowires‐based transistors including control and polarity gates is still in the concept phase. In fact, papers showing the combination of biological molecules and RFETs to realize “reconfigurable” biosensors were reported only a couple of years ago [273, 274, 275]. Here, the working principle is that the charges typically carried by biological molecules in physiological conditions affect the transistor conductivity similarly to an ISFET (ion‐sensitive Field Effect Transistor). However, the special property of RFETs, which allows them to operate as n‐type or p‐type devices, endows the reconfigurable biosensors with the ability to increase or decrease the channel current depending on the sign of the main charges of the biomolecules. In fact, the phosphate groups of any DNA or RNA molecules carry negative charges only, residues of amino acids in proteins may carry positive (in case of arginine, histidine, and lysine) or negative (in case of aspartic and glutamic acids) charges as well. Therefore, the probe biomolecule may have a net positive (e.g., the thrombin or an antibody) or a negative (e.g., a synthetic aptamer) charge in physiological solutions. Of course, the RFET may be biofunctionalized both in the region of the nanowire (e.g., in between the control and the program gates) [276] or on top of the control or on top of the program gates [277]. Therefore, a single RFET enables the design of biosensors with both positively and negatively charged probes, unlike ISFETs, where the net polarity of the target biomolecule must be clearly determined before the design stage.

The case of program gate bio‐functionalization is illustrated in Figure 14a. A negatively charged analyte, comprising the recognition element and the target molecule, results in *p*‐type behavior (Figure 14b), whereas a positively charged analyte yields *n*‐type conduction (Figure 14c). In the particular case of Ref. [277], the target molecule in the first case reduces hole conduction, lowering the magnitude of the on‐state current with increasing concentration, while the target molecule in the case of the *n*‐type conduction enhances the formation of the *n*‐type channel, increasing current, as seen in Figure 14d. The exact shape of the calibration curve (Figure 14d) depends on the complex interaction between the target molecule and the sensing element, and the resulting trends are not determined exclusively by the polarity of either. Thus, complex simulation and experimental characterization of the sensing element are required for each analyte combination.

**FIGURE 14:**
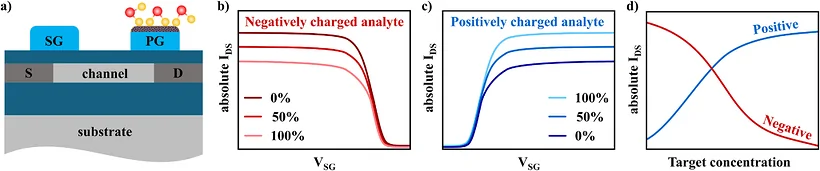
RFETs for biosensing. (a) Schematic of a DIG‐RFET bio‐functionalized in the program gate. When the polarity of the analyte (combination of target molecule and sensing element) (b) is negative, a *p*‐type channel is induced, whereas a (c) positively charged analyte induces an *n*‐type channel. (d) Calibration curves. In this case, an increase in concentration of the target molecule yields an increase in current for the positive analyte and a decrease in the case of the negatively charged analyte.

Beyond reconfiguring the sensing mechanism itself, RFET polymorphism can also be exploited at the circuit level to modify how multiple sensor outputs are processed. This is particularly relevant for multi‐target biosensing, where diagnostic information may depend not only on the detection of individual biomarkers but also on their combined logical relationships. In‐sensor computing approaches aim to perform such signal processing close to, or directly within, the sensing hardware, reducing the need for separate processing stages [278]. In Ref. [279], a circuit comprising nine TIG‐RFETs was proposed to process three biosensor inputs using the same reconfigurable hardware. By changing the RFET operating configurations and selectively including or bypassing individual sensing branches, different logic functions and combinations of the detected biomarkers can be evaluated without implementing separate dedicated logic circuits. Four operating modes were demonstrated through simulations, while up to eleven combinations of the three biochemical inputs were identified as accessible with the architecture. This illustrates how RFET reconfigurability can extend from adaptable transduction at the device level to adaptable information processing at the sensor interface.

### 5.4 Cryogenic Computing

Cryogenic control and readout circuits are indispensable for scaling quantum processors and potentially AI data centers, yet their power dissipation threatens to exceed the cooling capacity of dilution refrigerators. To this end, there is a need to integrate the readout and control circuitry in the cryogenic environment, either on‐chip or in close proximity to the computing units at relaxed cooling stages. At the same time. reducing the thermal load has become a fundamental requirement for viable quantum architectures. In this respect, reducing the power dissipation of control and readout transistors requires a decrease in the supply voltage to limit dynamic power consumption. Maintaining sufficient drive current at reduced supply voltages generally also requires lowering the threshold voltage, which in conventional transistors comes at the cost of increased subthreshold leakage and, consequently, higher static power. A substantially steeper subthreshold slope is therefore essential to preserve a favorable trade‐off between switching performance and static power at low operating voltages.

While millikelvin operation introduces unique semiconductor challenges, it also unlocks distinct advantages for emerging devices. Schottky‐barrier field‐effect transistors with lightly doped channels and metallic source/drain contacts, replacing the heavily doped source and drain regions of conventional MOSFETs, inherently exhibit fewer defect‐induced band‐tail states [280]. As a result, bandtail‐related leakage and switching degradation remain manageable rather than prohibitive. Furthermore, the altered, stronger body‐oriented transport, compared with the surface‐oriented transport in conventional inversion‐mode MOSFETs, can lead to superior noise characteristics [281]. Moreover, the Schottky contact may help mitigate direct source–drain tunneling, while the absence of heavy doping circumvents carrier freeze‐out, which typically degrades conventional devices at deep cryogenic temperatures [282]. However, the temperature‐dependent transport limitation at the injecting Schottky junctions needs to be addressed to ensure sufficiently large on‐currents $I_{\mathrm{on}}$.

The potential of RFETs for cryogenic control and readout circuits extends beyond these device‐level properties and can additionally be leveraged for architectural streamlining. As previously established, the multifunctionality of RFETs enables several conventional CMOS transistors to be replaced by a smaller number of reconfigurable devices. For a given circuit function, transistor‐count reductions of up to approximately 50% have been demonstrated [283]. This reduction in active components can translate into lower static and dynamic power consumption, thereby easing the cooling burden. RFET‐based circuits generally operate at lower frequencies than their CMOS counterparts because of the comparatively high resistance associated with carrier injection through the Schottky barriers. Nevertheless, their low off‐state leakage and reduced device count make them attractive for power‐constrained cryogenic applications, particularly for functions such as static biasing and multiplexing [284]. Furthermore, runtime reconfigurability can enable adaptive power gating and voltage scaling, allowing the circuit configuration and power envelope to be adjusted according to the required functionality [285].

The general functionality of RFETs at cryogenic temperatures is illustrated by simulating a nanowire‐based DIG‐RFET, shown in Figure 15a. In this device, the polarity gate function can be assigned to either the source gate (SG) or the drain gate (DG). When the SG acts as the polarity gate and the DG as the control gate—a configuration commonly referred to as program‐gate‐at‐source (PGAS)—the device exhibits an off‐state behavior similar to that of a conventional MOSFET with an ideal SS, but suffers from increased leakage (see the band diagram in Figure 15a). Measures that improve carrier injection through the Schottky barrier and thereby enhance the on‐state performance of the RFET, including ultrathin nanowire channels, gate‐all‐around geometries with high‐k gate dielectrics, and low‐band‐gap channel materials, typically lead to an exponential increase in this leakage [286, 287]. Conversely, when the DG acts as the polarity gate and the SG as the control gate—commonly referred to as program‐gate‐at‐drain (PGAD)—the leakage current is reduced at the cost of an increased SS due to the gate modulation of carrier injection through the Schottky barrier. At cryogenic temperatures, however, operation at small $V_{\mathrm{DS}}$, together with the reduced temperature, strongly suppresses this leakage and therefore enables operation with the SG acting as the polarity gate. Consequently, steep‐switching cryogenic RFETs can be envisioned by combining low‐band‐gap channel materials with optimized nanowire gate geometries.

**FIGURE 15:**
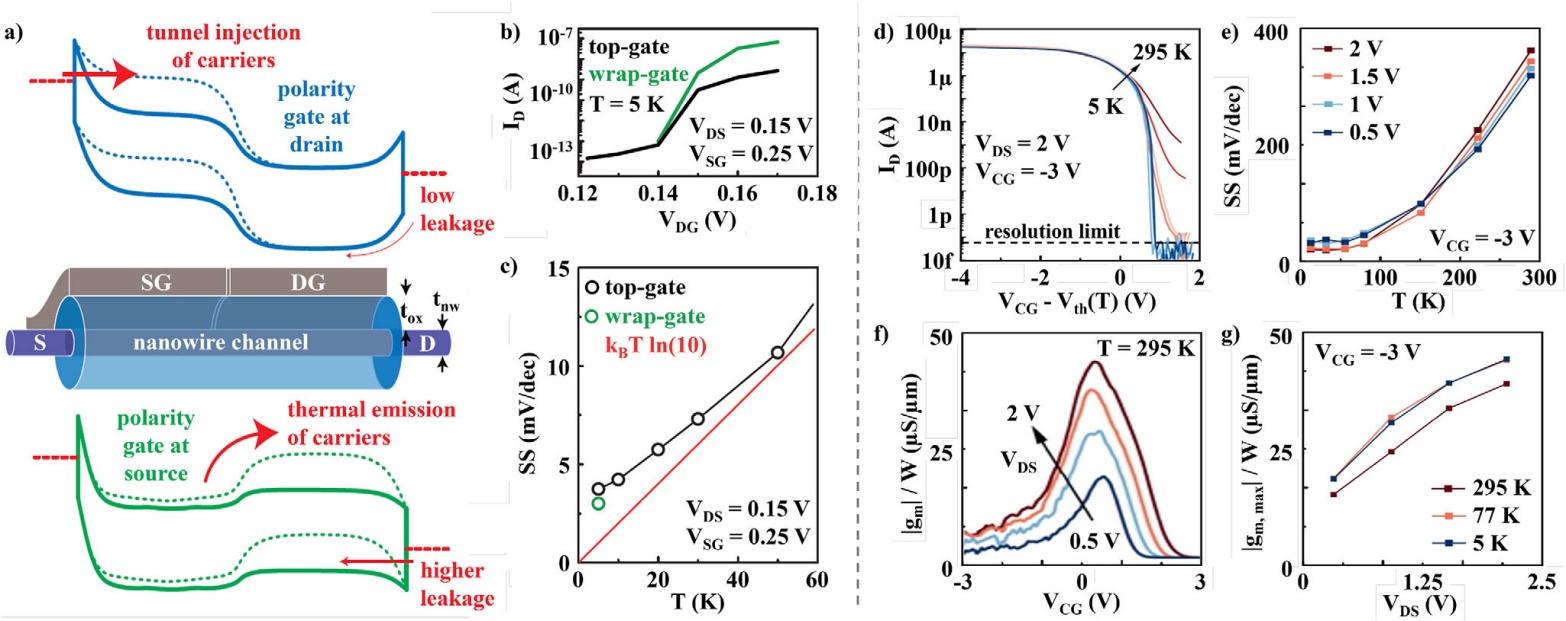
Cryogenic RFET operation. (a) Schematic of a nanowire DIG‐RFET with corresponding band diagrams of setting the polarity at the DG and SG. Self‐consistent simulations of a RFET set as *n*‐type at the SG showing (b) transfer characteristics at 5 K and (c) SS as a function of temperature of a top‐gate and a wrap‐gate nanowire DIG‐RFET with nanowire thickness of 2 nm, oxide thickness of 2 nm in case of top‐gate and 3 nm in case of wrap‐gate and a band gap of 0.3 eV. (d) Transfer characteristics of a SiGeSn channel TIG‐RFET set as p‐type measured at $V_{\mathrm{DS}}$ = 2 V between 5 and 295 K. (e) Extracted SS as a function of temperature for varying $V_{\mathrm{DS}}$ from 0.5 to 2 V. (f) Transconductance $g_{m}$ over temperature for a constant $V_{\mathrm{DS}}$ of 2 V. (g) Evaluation of $g_{m,\max}$ over temperature from 5 K up to 295 K for applying different $V_{\mathrm{DS}}$ from 0.5 to 2 V.

The operation of the device with the SG acting as the polarity gate is shown in Figure 15b. Two gate architectures are considered, both employing localized SG and DG electrodes along the nanowire. In the first configuration, the localized gates are positioned only on top of the nanowire. In the second, the same localized SG and DG sections wrap around the nanowire, forming a gate‐all‐around (GAA) architecture. The devices are simulated using a self‐consistent Poisson–Schrödinger solution within the non‐equilibrium Green's function formalism (for details on the simulations, see Ref. [288]). The GAA architecture increases the on‐state current by up to three orders of magnitude while maintaining a comparable leakage‐current level. Figure 15c shows the simulated SS as a function of temperature for the top‐gated architecture, together with the corresponding SS of the GAA architecture at 5 K. Both architectures enable steep switching at cryogenic temperatures, while the GAA implementation further improves the SS and on‐state performance without degrading the on/off ratio, as shown in Figure 15b. Combined with the transistor‐count reduction enabled by reconfigurability, these characteristics make steep‐switching RFETs a promising approach for reducing the power consumption of cryogenic control and readout circuits.

The operation of RFETs at cryogenic temperatures still requires rigorous experimental validation. Some studies have investigated multi‐gate control and temperature‐dependent transport without demonstrating complete polarity reconfiguration [289, 290]. More recently, full polymorphic switching has been demonstrated in an Al–Si material system down to 4.5 K [64], achieving SS values as low as 27 and 24 mV/dec for *p*‐ and *n*‐type operation, respectively. A key challenge for cryogenic RFET operation is the degradation of carrier injection and, consequently, the reduction in the on‐state current $I_{\mathrm{on}}$ and transconductance $g_{m}$ as the temperature decreases. Improved channel scaling, device geometry, and electrostatic gate control can enhance tunneling through the Schottky barrier, thereby mitigating this reduction. An additional strategy is to reduce the injection barrier itself, as demonstrated for a SiGeSn channel under cryogenic *p*‐type operation (see Figure 15d–g) [290]. In the TIG‐RFET architecture, quasi‐ohmic contacts can be achieved by simultaneously biasing the source‐ and drain‐side junction gates toward negative voltages. The reduced barrier strongly enhances hole injection and, with appropriate junction‐gate biasing, results in a peak $g_{m}$ that increases upon cooling.

Despite this progress, several steps are still required before the circuit‐level advantages of cryogenic RFETs can be established. In particular, the stability of electrostatic doping and Schottky‐barrier modulation must be assessed toward deep‐cryogenic and ultimately millikelvin temperatures, where changes in carrier statistics, quantum confinement, interface‐trap dynamics, and contact transport may alter device behavior. Circuit‐level benefits also remain to be experimentally demonstrated at low temperatures. Standard‐cell libraries and synthesis methodologies for RFETs are still under development even at room temperature [99, 106], and extending these approaches to cryogenic operation will require accurate temperature‐dependent models that capture threshold‐voltage shifts, Schottky‐barrier transport, and device variability. If cryogenic RFETs can be successfully engineered and validated, they would unlock profound circuit‐level advantages. The convergence of suppressed tunneling, minimal bandtail interference, and aggressive component reduction would directly translate into drastically lower power dissipation. Ultimately, bridging the gap between RFET innovation and cryogenic operation holds the key to transforming control and readout electronics from a thermal liability into a sustainable, energy‐efficient foundation for scalable quantum computing.

## 6 Opportunities and Challenges

### 6.1 Sustainability by Reliability and Reusability

Sustainability in modern electronic systems extends beyond energy efficiency to encompass environmental impact and hardware life cycle. This broader perspective is becoming increasingly important as the rapid evolution of computing workloads, particularly those associated with artificial intelligence, accelerates hardware obsolescence and contributes to the growing problem of electronic waste [291]. In this context, the inherent reconfigurability and tunability of RFETs offer a promising route toward extending the useful lifetime of electronic systems. At the circuit level, RFET‐based hardware can be reconfigured after fabrication, allowing the same physical platform to be repurposed for different computational tasks rather than being replaced when application requirements change. Such post‐fabrication adaptability could also facilitate the deployment of new or evolving algorithms on existing hardware. This is particularly relevant for AI, where algorithmic development often progresses faster than conventional hardware design and fabrication cycles. By enabling circuit functionality to be modified after fabrication, RFETs could therefore reduce the need for hardware replacement and contribute to more sustainable, longer‐lived electronic systems.

A second requirement for improving hardware sustainability is the extension of device lifetime through robust and reliable operation. While post‐fabrication reconfigurability can delay functional obsolescence, its benefits can only be fully exploited if RFETs remain stable under prolonged electrical stress. Accordingly, recent studies have investigated the degradation and aging mechanisms of RFETs under different operating conditions.

The ability of RFETs to dynamically switch between n‐ and p‐type conduction exposes a single device to stress conditions that are normally associated with different conventional MOSFET polarities. In particular, a RFET may experience both negative and positive bias temperature instability (NBTI and PBTI) during its lifetime. Early analyses indicate that NBTI has a more severe impact on threshold voltage degradation than PBTI, particularly when the device polarity is set as n‐mode. Furthermore, devices in which the SG acts as the polarity gate are vulnerable to subthreshold slope degradation. Hot carrier injection (HCI) also differs fundamentally from that of conventional CMOS devices. In MOSFETs, HCI‐induced damage is typically concentrated near the drain and is commonly associated with a reduction in on‐state current. In contrast, as a SB‐based device, the RFET features a different energy band profile, with the peak electric field occurring at the reverse‐biased source Schottky junction. Consequently, HCI‐induced damage is predominantly localized near the source and manifests primarily as a threshold‐voltage shift rather than a loss of $I_{\mathrm{on}}$. Hot‐electron stress has been found to affect *n*‐type operation more strongly than *p*‐type operation [292, 293, 294]. At the same time, the overall HCI‐induced degradation can be lower than in comparably scaled CMOS devices, indicating that RFETs may offer additional opportunities for extending device lifetime and improving the long‐term sustainability of electronic systems.

It is within this context that the unique architectural features of RFETs offer an additional route to extending system lifetime. The independent gate terminals of RFETs offer additional tuning compared to conventional CMOS technologies, which can be exploited to compensate for degradation‐induced parameter shifts. By adjusting the applied electrostatic potentials, changes in device characteristics caused by mechanisms such as BTI and HCI could, in principle, be counteracted to recover the desired operating point and performance without physically replacing the device [295, 296]. Such compensation would, however, require the bias conditions to be adapted throughout the device lifetime. This could be achieved through model‐based approaches, in which calibrated degradation models are used to periodically update the applied gate voltages, or through closed‐loop schemes in which in situ monitors detect performance degradation and provide feedback for adaptive bias control. Similar concepts are already employed in FD‐SOI CMOS technologies, where adaptive back‐gate biasing has been used to compensate process and aging‐induced variations [297, 298, 299]. The additional monitoring, bias‐generation, and control circuitry nevertheless introduces area and power overhead, and the influence of the compensating biases on leakage and further device degradation must therefore be considered. Ultimately, RFET technology could contribute to hardware that is not only repurposable for new tasks and adaptable to evolving algorithms but also capable of extending its operational lifetime through post‐fabrication electrical compensation.

### 6.2 Monolithic Back‐End‐Of‐Line Integration Compatibility

The device architecture required to enable RFET reconfigurability is inherently well‐suited to monolithic back‐end‐of‐line (BEOL) integration. BEOL integration imposes strict thermal constraints, typically below 400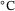, to avoid degradation of pre‐fabricated interconnects and underlying circuitry [300, 301]. RFETs employ undoped semiconductor channels and metallic source and drain regions, eliminating the need for complementary doping. Consequently, high‐temperature ion implantation and dopant activation anneals are not required, reducing the overall thermal budget. Device fabrication can therefore be realized using low‐temperature deposition techniques such as sputtering or atomic layer deposition. In addition, the lack of doping simplifies layer‐specific device engineering and variability associated with random dopant fluctuations, enabling cost‐effective sequential layer stacking in monolithic 3D integration [302, 303].

The electrostatic control of carrier polarity broadens the choice of channel materials by reducing the need for separate *n*‐ and *p*‐type doping. This is advantageous for the integration of materials such as polycrystalline silicon and other semiconductors compatible with low‐temperature processing. Moreover, low‐band‐gap materials can be integrated using BEOL‐compatible approaches such as layer transfer, van der Waals epitaxy on insulating substrates, or direct integration of two‐dimensional materials, partially mitigating performance trade‐offs associated with low thermal budgets [304, 305].

Multigate RFET architectures provide strong electrostatic control over the Schottky junctions and can suppress undesired carrier injection, thereby reducing off‐state leakage. This low‐leakage operation is particularly beneficial for memory arrays, logic‐in‐memory architectures, and BEOL digital logic, where limited heat dissipation and increasing power density impose stringent constraints on static power consumption [306, 307]. Combined with the reduced process complexity associated with doping‐free polarity control, these properties make RFETs promising candidates for densely integrated monolithic 3D systems. Especially noteworthy here is the opportunity to provide both p‐ and n‐type transistor functionality within a single element, thereby reducing integration costs. This positions monolithic BEOL processing as a future technology enabler for all circuit concepts discussed above.

### 6.3 The Challenge of System Scale‐Up

Most fabricated RFET hardware demonstrations to date have been limited to single devices or relatively small circuits, which remains insufficient to directly assess their true impact at the system level. At the same time, increasingly mature device models, standard‐cell libraries, synthesis methodologies, and architecture‐level studies have begun to enable system‐scale evaluation in simulation. In particular, complete RTL‐to‐GDSII implementation using RFET standard‐cell libraries compatible with an industrial 22 nm FDSOI technology has recently been demonstrated for the ISCAS'85 and EPFL'15 benchmark suites, providing one of the first realistic PPA assessments of RFET technology at larger circuit scales [99]. A more predictive PDK based on a 10 nm Silicon nanowire technology is also available [121]. Benchmarking tables for selected circuits in both technologies are shown in Table S2. To progress from such evaluations toward full system integration and fully exploit the benefits of RFETs in larger circuits, the electronic design automation (EDA) stack must undergo a paradigm shift that treats reconfigurability as a first‐class design dimension rather than a device‐level curiosity. Such developments can be broadly grouped into tightly coupled research directions spanning device‐level compact modeling, reconfiguration‐aware algorithms for logic and physical synthesis, EDA infrastructures capable of handling multi‐state behavior and enriched design–technology co‐optimization (DTCO), and system‐level management of the configuration itself.

First, accurate and efficient device modeling is the foundation of any large‐scale design using RFETs or related reconfigurable devices. Standard CMOS compact models, such as BSIM, do not capture dynamic polarity switching, dual‐carrier injection, or state‐dependent barrier profiles, and therefore cannot represent the added value and constraints of RFETs in circuit simulation. Early analyses of RFET‐based circuits had to rely on mixed‐mode simulation [98], simplified behavioral models, or logical‐effort style estimations [93], which do not scale to large designs or allow robust process‐voltage‐temperature (PVT) corner analysis. In contrast, SPICE‐compatible Verilog‐A [122, 155, 214, 308] and compact models [309, 310, 311, 312, 313] have recently become available for several RFET variants [314], including ferroelectric‐based RFETs and 2D‐material FETs, enabling DC, AC, and transient simulations within standard analog/mixed‐signal design flows. While these models capture key effects such as dual‐carrier injection and ferroelectric polarization dynamics, further work is needed to incorporate scalable transient behavior, process and geometry scaling, temperature dependence, stochastic variability, and noise, ideally supported by automated or machine‐learning‐based parameter extraction pipelines. Reliability also needs to become part of these models and characterization flows rather than being treated independently: first studies of bias‐temperature instability in TIG‐RFET devices and circuits already demonstrate that aging mechanisms must be propagated from device‐level degradation toward circuit‐level timing and functionality analysis [294].

Second, algorithms for logic and physical synthesis must be rethought to exploit RFET reconfigurability rather than treat RFET gates as fixed‐function CMOS drop‐in replacements. A single RFET‐based gate can implement multiple logic functions depending on the bias conditions [315], which opens additional degrees of freedom when mapping a Boolean network to a standard‐cell library. Early RFET‐specific technology mapping already demonstrated that explicitly exploiting the additional functionality of reconfigurable gates can outperform conventional CMOS‐oriented mapping strategies [316]. More recently, RFET‐specific synthesis has progressed beyond direct cell substitution toward logic representations that preserve properties intrinsic to the technology. In particular, Xor‐Majority Graph (XMG)‐based synthesis preserves the self‐duality naturally supported by RFET implementations of XOR and majority functions and has demonstrated area reductions of up to 12.36% compared with conventional synthesis approaches [97]. Synthesis approaches that consider reconfiguration from the outset—such as reconfigurable‐logic‐gate (RLG) aware technology mapping, exact synthesis, or SAT‐based multi‐function cell selection—have likewise been shown to deliver significantly better area and, in some cases, power than classical flows that ignore these capabilities [317].

To fully leverage RFETs, logic synthesis must co‐optimize functional mapping with configuration overhead, the cost of control networks, and potential benefits for obfuscation or hardware security. Similarly, physical synthesis steps such as placement and routing need to be adapted to incorporate additional program and control wiring, as well as potential security‐driven layout constraints, without negating the area gains of highly compact RFET cells through routing congestion and buffer insertion. Earlier work on double‐gate ambipolar Si nanowire FETs already recognized that the additional polarity‐control terminal creates a non‐trivial physical‐design challenge. To mitigate the resulting gate‐routing congestion, regular Sea‐of‐Tiles architectures were proposed in which prefabricated transistor‐pair tiles form the basis of the layout fabric [318, 319]. By co‐designing the symbolic layout and tile organization around the dual‐gate device structure, these approaches improved area utilization by up to approximately 16% relative to less suitable tile configurations, demonstrating that regular physical fabrics can partially absorb the routing overhead associated with reconfigurable devices. More recent RFET‐specific physical‐synthesis approaches extend this concept toward standard‐cell‐based design flows through optimized cell layouts, dedicated configuration‐driver cells, and configuration‐domain‐aware placement [320]. In this approach, larger inverter cells with high fan‐out are used to generate the polarity signal $V_{P}$ and its complement $\bar{V_{P}}$ for groups of reconfigurable cells, avoiding the need for an individual inverter within each cell. An island‐based placement strategy further exploits the Power‐Shut‐Off (PSO) functionality available in conventional place‐and‐route tools. Reconfigurable cells sharing the same $V_{P}/\bar{V_{P}}$ signals are grouped into dedicated power domains, while the driver cells are treated as power switches. Within each domain, the conventional local $V_{DD}$ and $V_{SS}$ rails are repurposed to distribute P and $\bar{P}$ to the reconfigurable cells, whereas the actual supply voltages are only required by the driver cells. This avoids dedicated routing resources for the polarity signals and reduces pin density within the reconfigurable island. Compared with a conventional physical‐synthesis treatment, such reconfiguration‐aware organization has demonstrated chip‐area reductions of up to 17.5% across benchmark circuits from the EPFL suite. This calls for reconfiguration‐aware cost functions and routing heuristics that explicitly trade off cell density, control wiring, timing closure, configuration fanout, and security objectives.

A related challenge that becomes increasingly important at the system scale is the distribution and management of the configuration itself. Reconfiguration cannot be considered a zero‐cost operation: program‐gate signals introduce additional capacitance and fanout, and early RFET circuit analyses already showed that the logical effort associated with a reconfiguration input can substantially exceed that of ordinary data inputs, making a function change slower than normal logic evaluation [104]. Moreover, in non‐reconfigurable optimized applications, a simple replacement of CMOS cells with their RFET counterparts can result in increased area when accounting for the reconfiguration overhead. At larger scales, the configuration network therefore becomes analogous to a dedicated control infrastructure that must distribute polarity states to potentially large numbers of devices or cells. The resulting reconfiguration latency and energy, the number of independently addressable configuration domains, and the storage and generation of configuration signals must be included in system‐level optimization. Depending on the application, it may be preferable to configure RFET blocks only infrequently, to share configuration drivers among groups of cells, or to exploit reconfiguration dynamically during operation. EDA tools will consequently need to distinguish between data‐path timing and configuration‐path timing and verify that transitions between configurations do not introduce unintended intermediate logic states.

Lastly, the broader EDA infrastructure needs to be upgraded to properly handle multi‐state logic and reconfigurable behavior across the full design flow. DTCO frameworks must be extended to explore RFET design spaces, identifying optimal device geometries, material stacks, operating voltages, circuit architectures, and degrees of reconfigurability for each application class (e.g., logic‐in‐memory, neuromorphic computing, hardware security). Initial technology/circuit co‐design frameworks have already demonstrated this concept by jointly sweeping RFET device parameters, such as supply voltage and nanowire dimensions and propagating their impact through standard‐cell characterization, logic synthesis, and physical design [321]. Such cross‐layer optimization is particularly important for RFETs because a device modification may simultaneously affect drive current, program‐gate efficiency, circuit topology, routing requirements, and the number of logic functions accessible from a cell.

Standard design formats, such as Liberty and LEF/DEF were originally conceived for fixed‐function standard cells and therefore need either to be enriched with explicit support for device polarity and configuration states or complemented by RFET‐specific abstractions describing state‐dependent timing, power, and functionality. Automated characterization flows must evolve to generate and manage the resulting increase in state‐dependent timing and power profiles while still remaining tractable for industrial‐scale libraries and PVT corners. Importantly, recent RFET standard‐cell work has demonstrated that conventional commercial EDA infrastructure can already be adapted to implement complete RTL‐to‐GDSII flows when appropriate RFET Liberty and physical‐library views are provided [99]. The remaining challenge is therefore not necessarily to replace the established EDA ecosystem, but to extend its abstractions and optimization engines so that reconfigurability itself participates in synthesis and design closure rather than being fixed before these steps.

Verification and sign‐off constitute another largely unexplored part of this problem. For a polymorphic cell, functional correctness must be established not only for all logical input combinations but also for every legal configuration state and, where runtime reconfiguration is used, during transitions between those states. Timing, power, variability, aging, and potentially security therefore become configuration‐dependent properties. Conventional corner‐based sign‐off could consequently grow rapidly with the number of independently configurable domains. Related combinatorial challenges are already encountered in conventional CMOS design, where multi‐corner multi‐mode timing analysis employs search‐space pruning and branch‐and‐bound techniques to avoid exhaustive scenario evaluation [322], formal verification relies on state‐space reduction to mitigate state explosion [323], symbolic switch‐level verification can represent families of input‐dependent circuit behaviors without explicit enumeration [324], and statistical timing analysis uses probabilistic representations, bounds, and selective enumeration to avoid exhaustive evaluation of process variations and timing scenarios [325, 326]. Similar concepts could therefore provide a starting point for RFET‐specific verification and characterization methodologies that avoid explicit characterization of every possible global configuration. RFET‐specific plugins for security, logic‐in‐memory mapping, reliability analysis, and configuration‐aware verification could ultimately be integrated into open‐source frameworks such as OpenROAD and LibreLane, providing accessible and transparent environments for RFET circuit development and enabling a broader community to experiment with reconfigurable technologies at realistic system scales.

Beyond ASIC‐oriented design flows, architecture‐level co‐design provides another route to translating device‐level reconfigurability into system benefits. RFET‐based FPGA architectures have recently been investigated using compact RFET LUT structures together with dedicated delay‐aware packing and partitioning algorithms. By co‐optimizing the device, circuit, architecture, and technology‐mapping layers, reductions of up to 39% in area, 36% in wire length, and 40% in switching energy relative to a conventional CMOS LUT‐based FPGA were reported [132]. Although these results remain simulation‐based, they demonstrate an important principle for RFET scale‐up: the largest gains may emerge only when the architecture and algorithms are redesigned around the device functionality rather than when RFETs are inserted into architectures originally optimized for CMOS. Similar cross‐layer approaches will likely be required for RFET‐based accelerators, logic‐in‐memory systems, and dynamically polymorphic hardware.

Beyond the individual technological challenges discussed above, system‐scale integration also raises the question of how RFETs should coexist with established CMOS technology. In the near term, heterogeneous integration appears to be the most realistic approach. Conventional CMOS can provide high‐drive and fixed‐function circuitry, while RFETs can be selectively introduced in blocks where polarity control, tunability, or multi‐functionality provide a clear advantage. Such hybrid systems also reduce the burden on the RFET design ecosystem, since clock generation, memory, I/O, power management, and other mature infrastructure can initially remain CMOS‐based while RFETs are deployed only where their additional functionality compensates for their current performance or integration penalties. Determining this partition automatically represents an additional system‐level optimization problem: future EDA flows could select not only which RFET function should implement a logic node, but also whether that node should be implemented using RFET or CMOS technology at all.

BEOL integration offers an additional route toward such heterogeneous systems: the lightly doped or doping‐free semiconductor regions and Schottky‐barrier contacts required for RFET operation relax the dependence on high‐temperature implantation and dopant activation, potentially allowing reconfigurable functionality to be vertically integrated above conventional CMOS without occupying additional FEOL transistor area. Fully RFET‐based systems may nevertheless become attractive for strongly power‐ or area‐constrained applications in which functional density and adaptability outweigh maximum switching performance. In the longer term, the integration of low‐band‐gap and low‐dimensional channel materials, for which conventional complementary doping can be challenging, could further shift this balance by reducing the performance penalty typically associated with Schottky‐barrier injection. Lastly, new approaches to fine‐grained device reconfiguration, such as the dual‐doped RFET principle suggested in simulations [162, 245], might open avenues for utilizing fine‐grained reconfiguration in high‐performance electronic systems.

## 7 Conclusion

This paper presents a comprehensive review of the state‐of‐the‐art in RFET circuit design, starting with the underlying physical properties that RFETs provide, including their embodiments on a variety of material systems. Functional enhancement can be achieved by operating the device in one of four conduction modes: *n*‐type, *p*‐type, ambipolar, or anti‐ambipolar. In addition, the multigate structure of RFETs can be used to tune their device parameters to match the individual application. Despite having similar structures and operating principles, we have shown that group IV channel materials still outperform layered 2D materials in terms of performance per unit applied voltage. Key achievements in digital, analog, and unconventional computing are reviewed and exemplified by new measurements on a 22 nm FDSOI technology. Hardware security and edge computing with post‐Shannon data processing approaches are identified as the two areas in which the underlying properties of RFETs are best positioned to deliver the greatest system‐level benefit. The relatively new application areas of bio‐ and optical sensing and cryo‐electronics for quantum interfacing are introduced as potential avenues for future development. The paper concludes with an outlook on the possibilities and challenges regarding reliability, monolithic back‐end‐of‐line integration, and electronic design automation needed for system‐scale‐up of future reusable and sustainable electronics. Overall, our work illustrates that RFETs are a unique class of emerging devices that have begun to outgrow the lab‐based development stage. Future work will need to focus more strongly on the application‐driven design of circuits and systems that incorporate RFET technology, thereby rethinking how best to exploit the inherently higher functionality of reconfigurable transistors.

## 8 Experimental Section

The RFET test structures used in this work are fabricated on 300 mm wafers using GlobalFoundries 22 nm technology with minor modifications to the standard baseline CMOS process. The integration flow includes a well contact formation to electrically connect the back gate (BG) below the buried‐oxide, a gate‐first high‐k metal gate (HKMG) stack, a set of spacers, and the complete BEOL. The source and drain terminals have been modified to create silicide contacts reaching into the doping‐free channel regions below the outer gates, forming Schottky junctions. A design solution was developed to avoid the need for additional masks and prevent silicide formation between the individual gates of a single device. Rather large designs have been chosen to ensure a higher device yield in the early phase of industrial demonstration.

All measurements were performed directly on the wafer using a fully‐automatic probe station (UF3000 from ACCRETECH). Quasi‐stationary measurements are employed to mimic the waveform and verify logic functionality through complete truth‐table evaluation. These measurements were carried out using a Keithley 4200A‐SCS parameter analyzer equipped with source measurement unit (SMU) modules. The output terminal is current‐biased with an SMU to avoid pinning at a particular voltage level.

### 8.1 Second Part of Experimental Section

Grammarly, ChatGPT, and Gemini were used in the preparation of this manuscript for text editing, grammar checking, and knowledge retrieval. No passages were copied without full author review, and all substantive ideas, analysis, and conclusions are the product and responsibilities of the authors. All figures are original without the involvement of AI tools.

## Conflicts of Interest

The authors declares no conflicts of interest.

## Supporting information


**Supporting File**: advs78121‐sup‐0001‐SuppMat.pdf.

## Data Availability

The data that support the findings of this study are available from the corresponding author upon reasonable request.
