# Supplementary material for: Re‐Thinking Electronic Circuit Design Through Emerging Reconfigurable Field Effect Transistors
Source: Adv Sci (Weinh). 2026 Oct 14:e78121. Online ahead of print. doi: 10.1002/advs.78121 (PMC13668769; doi:10.1002/advs.78121)
Supplement: Supplementary file 1 [file ADVS-9999-e78121-s001.pdf]

Re-Thinking Electronic Circuit Design Through Emerging Reconfigurable Field Effect Transistors

Jens Trommer [1] *Juan P. Martinez* [1,2] *Yuxuan He* [1,2] *Giulio Galderisi* [1] *Katrin Pingen* [1,2] *Eduardo Sergio Oliveros-Mata* [1] *Lisa Tellis* [1] *Cigdem Cakirlar* [1] *Christoph Beyer* [1] *Brojogopal Sapui* [1] *Joachim Knoch* [3] *Klaus Hofmann* [4] *Sandro Carrara* [5] *Eugenio Cantatore* [6] *Max C. Lemme* [3,7] *Ian O'Connor* [8] *Akash Kumar* [9] *Andre Heinzig* [2] *Thomas Mikolajick* [1,2] *Walter M. Weber* [10]

[1] Namlab gGmbH, Dresden, Germany
[2] TU Dresden, Dresden, Germany
[3] RWTH Aachen, Aachen, Germany
[4] TU Darmstadt, Darmstadt, Germany
[5] EPFL, Lausanne, Switzerland
[6] TU Eindhoven, Eindhoven, Netherlands
[7] AMO GmbH, Aachen, Germany
[8] Ecole Central Lyon, Lyon, France
[9] Ruhr-University Bochum, Bochum, Germany
[10] TU Wien, Vienna, Austria
E-mail Address: jens.trommer@namlab.com, thomas.mikolajick@tu-dresden.de, walter.weber@tuwien.ac.at

Keywords: *reconfigurable field effect transistors, van-der-Waals materials, circuit design, hardware security, logic-in-memory, bio-sensors, cryogenic temperatures*

Reconfigurable field-effect transistors, short RFETs, are a unique class of emerging electronic devices that merge n-type, p-type and ambipolar operation modes into a single element. Unlike conventional MOSFETs, the dominant carrier type in RFETs is purely defined by electrostatics and can be dynamically changed at runtime. This unlocks a variety of new design possibilities by enabling post-fabrication functionality definition, thereby increasing circuit density and adaptability without requiring additional feature size scaling. However, the added value provided by the devices requires a reassessment of classical design paradigms in order to unfold their full potential. This paper aims to give a comprehensive review of the state-of-the-art in RFET circuit design. Starting from the underlying device physics and material systems, such as Si, Ge, 2D materials, and carbon nanotubes, the manifold circuit design possibilities in digital, analog, and neuromorphic computing are introduced. We highlight how RFETs aim to enable simplified, energy-efficient, and reusable hardware for edge computing, hardware security, bio-sensing, and cryogenic electronics. Thereby, both device-and circuit-level results are exemplified by measurements of Three-Independent-Gate (TIG)-RFETs processed on an industrial 22nm FDSOI platform as well as SiGe devices fabricated in a lab environment. The paper also provides an overview of future key challenges in reliability, back-end-of-line integration, and electronic design automation, offering an outlook on the path towards scalable, sustainable reconfigurable electronics.

1 Low-Power Digital Circuits: Truth tables

a)

A1	A0	B1	B0	Cin	Result (Cout S1 S0)
0	0	0	0	0	0 0 0
0	0	0	0	1	0 0 1
0	0	0	1	0	0 0 1
0	0	0	1	1	0 1 0
0	0	1	0	0	0 1 0
0	0	1	0	1	0 1 1
0	0	1	1	0	0 1 1
0	0	1	1	1	1 0 0
0	1	0	0	0	0 0 1
0	1	0	0	1	0 1 0
0	1	0	1	0	0 1 0
0	1	0	1	1	0 1 1
0	1	1	0	0	0 1 1
0	1	1	0	1	1 0 0
0	1	1	1	0	1 0 0
0	1	1	1	1	1 0 1
1	0	0	0	0	0 1 0
1	0	0	0	1	0 1 1
1	0	0	1	0	0 1 1
1	0	0	1	1	1 0 0
1	0	1	0	0	1 0 0
1	0	1	0	1	1 0 1
1	0	1	1	0	1 0 1
1	0	1	1	1	1 1 0
1	1	0	0	0	0 1 1
1	1	0	0	1	1 0 0
1	1	0	1	0	1 0 0
1	1	0	1	1	1 0 1
1	1	1	0	0	1 0 1
1	1	1	0	1	1 1 0
1	1	1	1	0	1 1 0
1	1	1	1	1	1 1 1

b)

S0	S1	S2	Out 1	Out 2
0	0	0	XOR	AND
0	0	1	XNOR	OR
0	1	0	NAND	AND
0	1	1	NOR	OR
1	0	x	3XOR	3MAJ
1	1	x	3MIN	3MAJ

c)

X4	X3	X2	X1	Carry	Sum	ED
0	0	0	0	0	0	0
0	0	0	1	0	1	0
0	0	1	0	0	1	0
0	0	1	1	0	1	-1
0	1	0	0	1	0	1
0	1	0	1	1	0	0
0	1	1	0	1	1	0
0	1	1	1	1	0	0
1	0	0	0	1	0	1
1	0	0	1	1	0	0
1	0	1	0	1	0	0
1	0	1	1	1	1	-1
1	1	0	0	1	1	1
1	1	0	1	1	1	0
1	1	1	0	1	1	0
1	1	1	1	1	1	-1

Figure S1: Truth tables corresponding to the RFET circuit implementations presented in the main text. a) 2-bit full adder utilizing higher-order 3XOR and 3MAJ gates. b) Reconfigurable ALU utilizing gate-level reconfiguration. c) Approximate 4:2 compressor with reduced gate complexity. For the approximate compressor, ED denotes the Error Distance, defined as the difference between the numerical value of the approximate output and the exact arithmetic sum of the four inputs. Accordingly, a positive ED indicates that the approximate result overestimates the exact result, whereas a negative ED indicates an underestimation; ED = 0 corresponds to an exact output.

2 State-of-the-art Benchmarking

Table S1: Benchmark of RFET-based standard cells in two different implementations, namely FDSOI and SiNW, against comparable CMOS tecnologies such as 22nm FDSOI and 7nm FinFET.

**Cell**<br>Metric	FDSOI<br>**TIG-RFET** [A]	22nm FDSOI<br>CMOS [A]	10nm SiNW<br>**TIG-RFET [B]**	7nm FinFET<br>CMOS [C]
**NOT**				
Area (μm^2)	1.45	1.1	0.072	0.05
Delay (ns)	2.4	0.003	0.197	0.007
Total Power (nW)	N.A.	N.A.	N.A.	0.142
Leakage Power (pW)	0.4	1000	37	100
**NAND2**				
Area (μm^2)	1.98	1.28	0.17	0.08
Delay (ns)	3.4	0.004	0.258	0.013
Total Power (nW)	N.A.	N.A.	N.A.	0.124
Leakage Power (pW)	0.2	1053	19.2	130
**NOR2**				
Area (μm^2)	1.98	1.28	0.22	0.06
Delay (ns)	3.7	0.004	0.223	0.014
Total Power (nW)	N.A.	N.A.	N.A.	0.08
Leakage Power (pW)	0.4	1027	36.8	N.A.
**XOR2**				
Area (μm^2)	3.02	1.98	0.345	0.087
Delay (ns)	6.3	0.006	0.532	0.087
Total Power (nW)	N.A.	N.A.	N.A.	N.A.
Leakage Power (pW)	1.3	3987	135.5	N.A.

Table S2: Metrics from ISCAS-85 benchmark circuits in two different RFET implementations, namely FDSOI and SiNW, against comparable CMOS tecnologies such as 22nm FDSOI, 10nm GAA, and 7nm FinFET.

**Circuit**<br>Metric	FDSOI<br>**TIG-RFET** [A]	22nm FDSOI<br>CMOS [A]	10nm SiNW<br>**TIG-RFET** [B]	10nm GAA-NW<br>CMOS [D]	7nm FinFET<br>CMOS [E]
**c432**					
Area (μm^2)	230.95	134.32	21.37	12.6	6.76
Delay (ns)	242	0.234	12.9	N.A.	0.081
Total Power (nW)	0.51	125.93	5.83	2980	8660
**c880**					
Area (μm^2)	493.81	324.9	48.56	22	15.3
Delay (ns)	278	0.219	12.1	N.A.	0.075
Total Power (nW)	0.99	326.86	13.9	5380	8440
**c3540**					
Area (μm^2)	1475.63	956.82	143.4	59.6	35.6
Delay (ns)	357	0.294	20.81	N.A.	0.118
Total Power (nW)	3.65	978.68	44	25600	17590

3 References Supplemental Material

[A] Kavand, Nima, et al. "Design and Evaluation of an RFET Standard Cell Library Compatible with 22 nm FDSOI." IEEE Transactions on Computer-Aided Design of Integrated Circuits and Systems, vol. 45, no. 9, pp. 4072-4085, 2026.
[B] Gauchi, Roman, Ashton Snelgrove, and Pierre-Emmanuel Gaillardon. "An open-source three-independent-gate fet standard cell library for mixed logic synthesis." 2022 IEEE International Symposium on Circuits and Systems (ISCAS). IEEE, 2022.
[C] Lakshmanan, Anirudh, Gaurav Kumar Mishra, and Tunga Ravi. "Design and analysis of 7nm FinFET full custom standard cell library using ASAP7 PDK." 2022 6th International Conference on Computing Methodologies and Communication (ICCMC). IEEE, 2022.
[D] Wang, Luhao, et al. "Standard cell library based layout characterization and power analysis for 10nm gate-all-around (GAA) transistors." 2016 29th IEEE International System-on-Chip Conference (SOCC). IEEE, 2016.
[E] Xie, Qing, et al. "Performance comparisons between 7-nm FinFET and conventional bulk CMOS standard cell libraries." IEEE Transactions on Circuits and Systems II: Express Briefs 62.8: 761-765, 2015.

4 Acknowledgements

This work is co-funded by the German Research Foundation (DFG) under TRR404 Active-3D project Nb.528378584, by the German Ministry for Research, Technology and Space (BMFTR) under the framework DI-ReDesign, by the European Union under the HORIZON framework (Nb. 101135316), and from tax revenues based on the budget adopted by the Saxon State Parliament. GlobalFoundries Fab 1 in Dresden is acknowledged for the fabrication of the FDSOI-based devices presented in this work. Katrin Pingen, Christoph Beyer, and Lisa Tellis acknowledge by a cooperation co-funded by Infineon Technologies Dresden AG & Co. KG within the course of IPCEI ME/CT. Nima Kavand from Ruhr University Bochum, Germany, is acknowledged for generating the benchmark data for 10 nm SiNW TIG-RFETs in the supplemental information. Views and opinions expressed are, however, those of the authors only and do not necessarily reflect those of the European Union. Neither the European Union nor the granting authority can be held responsible for them.
